# RAD21 amplification epigenetically suppresses interferon signaling to promote immune evasion in ovarian cancer

**DOI:** 10.1172/JCI159628

**Published:** 2022-11-15

**Authors:** Peng Deng, Zining Wang, Jinghong Chen, Shini Liu, Xiaosai Yao, Shaoyan Liu, Lizhen Liu, Zhaoliang Yu, Yulin Huang, Zhongtang Xiong, Rong Xiao, Jiuping Gao, Weiting Liang, Jieping Chen, Hui Liu, Jing Han Hong, Jason Yongsheng Chan, Peiyong Guan, Jianfeng Chen, Yali Wang, Jiaxin Yin, Jundong Li, Min Zheng, Chao Zhang, Penghui Zhou, Tiebang Kang, Bin Tean Teh, Qiang Yu, Zhixiang Zuo, Qingping Jiang, Jihong Liu, Ying Xiong, Xiaojun Xia, Jing Tan

**Affiliations:** 1 Sun Yat-sen University Cancer Center, State Key Laboratory of Oncology in South China, Collaborative Innovation Center for Cancer Medicine, Guangzhou, Guangdong, China.; 2 Institute of Molecular and Cell Biology, Singapore.; 3 The Third Affiliated Hospital of Guangzhou Medical University, Guangzhou, Guangdong, China.; 4 Guangdong Provincial Key Laboratory of Colorectal and Pelvic Floor Diseases, The Sixth Affiliated Hospital of Sun Yat-sen University, Guangzhou, Guangdong, China.; 5 Cancer and Stem Cell Biology Program, Duke-NUS Medical School, Singapore.; 6 Laboratory of Cancer Epigenome, Division of Medical Sciences, National Cancer Centre Singapore, Singapore.; 7 Genome Institute of Singapore, Agency for Science, Technology and Research (A*STAR), Singapore.; 8 Cancer Science Institute of Singapore, National University of Singapore, Singapore.; 9 SingHealth/Duke-NUS Institute of Precision Medicine, National Heart Centre Singapore, Singapore.

**Keywords:** Immunology, Oncology, Cancer immunotherapy, Epigenetics, Genetic instability

## Abstract

Prevalent copy number alteration is the most prominent genetic characteristic associated with ovarian cancer (OV) development, but its role in immune evasion has not been fully elucidated. In this study, we identified *RAD21*, a key component of the cohesin complex, as a frequently amplified oncogene that could modulate immune response in OV. Through interrogating the RAD21-regulated transcriptional program, we found that RAD21 directly interacts with YAP/TEAD4 transcriptional corepressors and recruits the NuRD complex to suppress interferon (IFN) signaling. In multiple clinical cohorts, RAD21 overexpression is inversely correlated with IFN signature gene expression in OV. We further demonstrated in murine syngeneic tumor models that RAD21 ablation potentiated anti–PD-1 efficacy with increased intratumoral CD8^+^ T cell effector activity. Our study identifies a RAD21–YAP/TEAD4–NuRD corepressor complex in immune modulation, and thus provides a potential target and biomarker for precision immunotherapy in OV.

## Graphical abstract

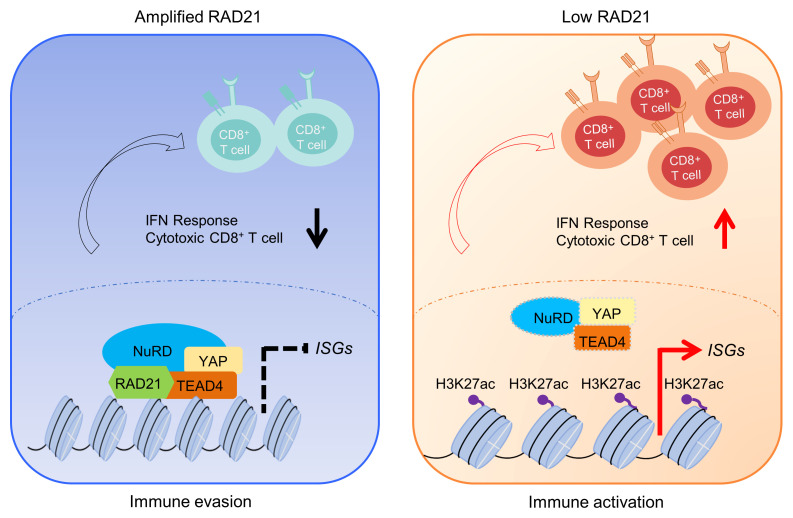

## Introduction

Ovarian cancer (OV) is the most lethal cancer among gynecological malignancies, largely owing to late diagnosis and resistance to chemotherapy (1), motivating research efforts to exploit new therapeutic options. Immune checkpoint inhibitors, including anti–programmed cell death 1 (anti–PD-1) antibody and anti–programmed cell death ligand 1 (anti–PD-L1) antibody, have shown remarkable clinical benefits in certain cancer types, such as melanoma and lung cancer (2, 3), but have limited efficacy in OV (4). High-grade serous ovarian cancer (HGSOC) has the worst prognosis and is the deadliest subtype of OV, accounting for 70%–80% of OV deaths. The primary genetic features of HGSOC include *TP53* mutations (96%) and prevalent genomic copy number alterations (CNAs) (5). CNA is essential for genome instability and has been recognized as a hallmark in multiple human cancers (6). Increasing evidence has revealed the role of CNAs in regulating antitumor immunity. For instance, amplification of *PRKCI* promoted immune suppression in OV (7). In melanoma patients, recurrent *G9a* gene amplification was associated with immune evasion (8). In addition, amplification of *SETDB1* was associated with immune exclusion in human tumors (9). These findings suggested that CNAs play crucial roles in antitumor immunity of human tumors, raising the possibility that targeting CNA genes may represent a novel therapeutic strategy for OV treatment.

Cohesin is an evolutionarily conserved complex that consists of 4 core subunits, RAD21, STAG1/2, SMC1A, and SMC3, and is required for chromosome segregation, homologous recombination, and maintenance of genome stability (10). Deregulation of cohesin frequently occurs in various types of human cancer, including solid tumors and hematological malignancies (11). For instance, frequent somatic mutations of *STAG2*, *RAD21*, *SMC1A*, and *SMC3* were reported in different myeloid neoplasms and bladder cancer (12, 13). Truncating mutation of *STAG2* was found in glioblastoma, melanoma, and urothelial bladder cancer (14, 15). Among these genes, RAD21 is one of the most critical components of the cohesin complex, encoding a DNA double-strand-break repair protein (16). RAD21 plays critical roles in multiple developmental processes and transcriptional programs. Canonically, RAD21 and the insulator-binding protein CTCF bind to highly conserved promoters and distant enhancers, contributing to transcriptional regulation (17). Spatial transcriptomics have shown that RAD21 organizes chromatin loops at replication factories, thereby facilitating simultaneous firing of the clustered origins (18). RAD21 is also important for maintenance of embryonic stem cell identity through cooperating with the key pluripotency transcription factors, such as Nanog and Sox2 (19, 20). Furthermore, recent reports have highlighted the dual role of RAD21 in transcriptional regulation, which functions as a corepressor through polycomb repressive complex 2 (PRC2) or as a coactivator with MYC to regulate expression of downstream targets (21, 22). In addition, aberrant function of RAD21 has been implicated in multiple cancer types, including breast cancer, colon cancer, and myeloid leukemia, and is correlated with malignant progression and poor prognosis (23–25). Such studies highlight the oncogenic role of RAD21 in driving tumorigenesis in various human cancers. However, whether genomic alterations of *RAD21* affect antitumor immune responses remains to be explored.

In this study, we seek to unravel novel CNA genes that are functionally linked to antitumor immune responses, which may provide new therapeutic targets to improve clinical outcomes in HGSOC. Our study revealed that *RAD21* gene amplification can promote immune evasion in OV and does so by suppressing the interferon (IFN) signaling pathway via interaction with the YAP/TEAD4 corepressor complex. Importantly, RAD21 ablation induced T cell activation and enhanced antitumor efficacy of anti–PD-1 in multiple murine syngeneic tumor models.

## Results

### RAD21 is a critical CNA gene and amplified RAD21 correlates with poor prognosis in HGSOC.

**Figure 1:**
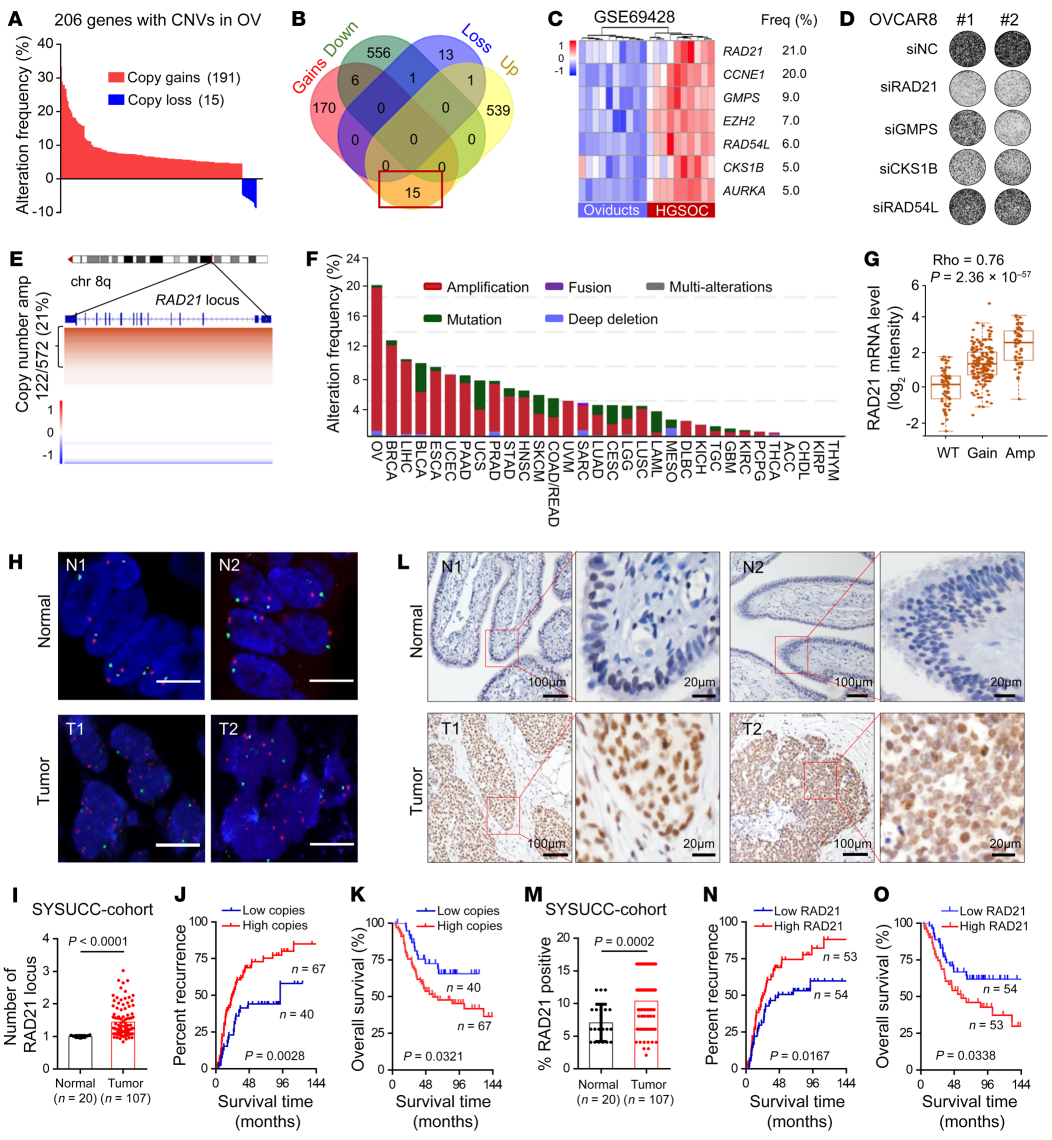
RAD21 is a critical CNA gene and amplified RAD21 correlates with poor prognosis in HGSOC. (**A**) Bar graph showing the CNA frequencies (191 copy gains and 15 copy loss) in the TCGA-OV database. (**B**) Venn diagram showing the overlap of 206 CNAs and DEGs in human OV in the TCGA database (TCGA-OV transcriptome_U133A-seq database, adjusted *P* value < 0.01, |log_2_foldchange| > 1). (**C**) Heatmap for upregulated genes (adjusted *P* value < 0.01) in 10 pairs of independent patients from GSE69428 database. (**D**) Representative images of colony formation assay in OVCAR8 cells transfected with scramble siRNA or 2 individual siRNAs targeting *RAD21*, *GMPS*, *CKS1B*, and *RAD54L*, respectively. (**E**) Integrative Genomics Viewer heatmap displays the CNAs on the *RAD21* locus, obtained from data of OV in cBioportal (*n* = 572; copy number amplification was defined as copy number score > 0.3). (**F**) Genetic alteration frequency of *RAD21* in TCGA pan-cancer database. (**G**) Correlation analysis showing an increase in *RAD21* mRNA level concordant with the gain of an additional DNA copy (Gain) and/or multiple copies (Amp) (Spearman ρ = 0.76; *P* = 2.36 × 10^–57^). (**H** and **I**) Representative images (**H**) and quantification (**I**) of FISH showing *RAD21* locus in ovarian tumor (*n* = 107) and normal fallopian tube tissue (*n* = 20) from SYSUCC cohort. Blue, DNA stained with DAPI; green, centromere of chromosome 8; red, genomic locus of *RAD21* gene. Scale bars: 5 μm. (**J** and **K**) Kaplan-Meier curves of recurrence time (**J**) and overall survival rates (**K**) in patients with OV grouped according to high (red, *n* = 67) and low (blue, *n* = 40) copies of *RAD21* (log-rank test). (**L** and **M**) Representative IHC staining (**L**) and quantification (**M**) showing RAD21 expression in ovarian tumor (*n* = 107) and normal fallopian tube tissue (*n* = 20) from SYSUCC cohort. (**N** and **O**) Kaplan-Meier curves of recurrence time (**N**) and overall survival rates (**O**) in patients with OV grouped according to high (red, *n* = 53) and low (blue, *n* = 54) expression of RAD21 (log-rank test). Data in **I** and **M** are shown as mean ± SD (2-tailed *t* test).

Prevalent somatic copy number alteration (CNA) is a characteristic feature of the HGSOC genome (26), suggesting that CNA genes may contribute to tumorigenesis of HGSOC. To identify the critical CNA genes, we systematically assessed the frequency of CNA using 572 profiled samples from the TCGA (The Cancer Genome Atlas) database (26). To discover and identify the potential driver genes with CNA, a total of 206 genes with a frequency of CNA in more than 5% of OV patients were selected, including 191 amplified genes (copy number gains) and 15 deleted genes (copy number loss) (Figure 1A and Supplemental Table 1; supplemental material available online with this article; https://doi.org/10.1172/JCI159628DS1). Among these genes, 15 had copy number gains and were upregulated in ovarian tumors compared with normal ovary by pairwise comparison analysis between microarray and CNA database in TCGA (Figure 1B). Seven of these 15 amplified genes were significantly upregulated in an independent cohort of HGSOCs (*n* = 10) compared with matched normal oviduct samples (GSE69428, Gene Expression Omnibus database) (Figure 1C) (27), including *AURKA*, *CCNE1*, and *EZH2*, which are known driver genes in HGSOC (28–30). The remaining 4 genes have not previously been studied and may serve as potential driver genes in HGSOC. To determine the potential roles of these 4 candidates, we performed siRNA-mediated silencing in OVCAR8 cells. Targeting of *GMPS*, *CKS1B*, and *RAD21* by 2 individual siRNAs attenuated colony-formation potential of cancer cells, while depletion of *RAD54L* failed to exhibit any effect on colony formation (Figure 1D and Supplemental Figure 1A). Notably, *RAD21* was the most frequently amplified and overexpressed gene in OV (Figure 1C), suggesting that *RAD21* may function as a novel key driver gene for this disease.

Analysis of genomic alterations of OV from TCGA data showed that copy number amplification at the *RAD21* locus was detected in 21% of OV patients (122/572) (Figure 1E). Pan-cancer analysis of TCGA data revealed that *RAD21* amplification occurs most frequently in OV (21%) compared with 31 other cancer types (Figure 1F). In addition, HGSOC patients harbor increased *RAD21* somatic copy number with a significant positive correlation between *RAD21* transcript abundance and somatic copy number alterations (Spearman ρ = 0.76; *P* = 2.36 × 10^–57^) (Figure 1G). Kaplan-Meier survival analysis (http://kmplot.com/analysis) showed that high *RAD21* expression was associated with worse progression-free survival (*P* = 0.0011) (Supplemental Figure 1B) (31), and especially correlated with poor prognosis in the early stage of HGSOC (*P* = 0.0031) (Supplemental Figure 1C), suggesting that RAD21 may drive tumor initiation in HGSOC.

To further investigate the clinical significance of *RAD21*, we first performed a correlation analysis between DNA copy number and expression of *RAD21* in patient-derived cells and OV cell lines and found that the mRNA level of *RAD21* was positively correlated with its DNA copy number (Supplemental Figure 1, D and E). We next examined the copy number alteration of *RAD21* using fluorescence in situ hybridization (FISH) in 107 cases of HGSOC and 20 cases of normal fallopian tube tissues from Sun Yat-sen University Cancer Center (SYSUCC cohort). The FISH data showed that *RAD21* chromosomal locus was amplified in HGSOC cases compared with normal fallopian tube tissues (Figure 1, H and I). In addition, *RAD21* amplification was significantly associated with worse relapse-free survival (*P* = 0.0028) (Figure 1J) and overall survival (*P* = 0.0321) (Figure 1K). We also examined RAD21 protein expression using immunohistochemistry (IHC) in the same cohort. Consistently, RAD21 was overexpressed in HGSOC relative to normal tissues (Figure 1, L and M), concordant with its amplification, which led to a poor prognosis (Figure 1, N and O, and Supplemental Figure 1F). The association of high RAD21 expression with lower survival was also observed in multiple cancer types, including adrenocortical carcinoma, bladder urothelial carcinoma, cervical squamous cell carcinoma, lung squamous cell carcinoma, mesothelioma, and sarcoma (Supplemental Figure 1, G–L). Taken together, these data suggest that amplified *RAD21* may play a pivotal role in tumorigenesis of HGSOC, functioning as a potential CNA driver gene.

### Genome-wide profiling analysis identifies repression of IFN genes by RAD21 and its associated repressor complexes.

**Figure 2:**
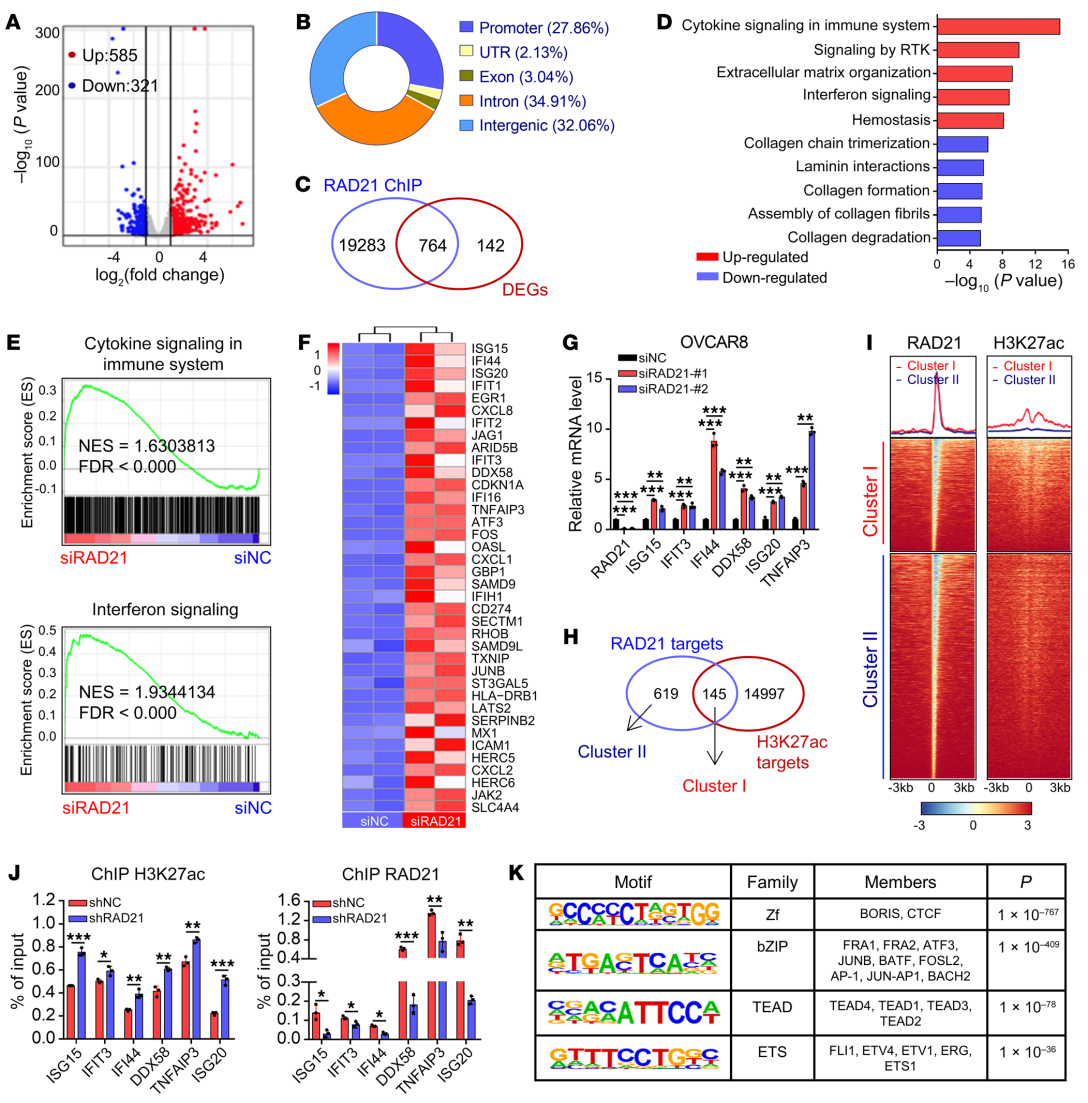
Genome-wide identification of potential targets of RAD21 and its associated cohesin complexes. (**A**) Scatterplot of DEGs between *RAD21*-knockdown (*RAD21*-KD) and control OVCAR8 cells (duplicates, *P* < 0.05, |log_2_foldchange| > 1). (**B**) Genomic distribution of RAD21 peaks in OVCAR8 cells. (**C**) Venn diagram showing the overlaps of DEGs and RAD21 direct targets obtained from ChIP-Seq results. (**D**) The 764 DEGs described in **C** were functionally clustered using the RECTOME gene sets. The top 5 upregulated and downregulated pathways are shown. (**E**) GSEA analysis showing that Cytokine Signaling in Immune System and IFN Signaling were enriched among the upregulated pathways. NES, normalized enrichment score. (**F**) Heatmap for significantly upregulated IFN signaling genes (*P* < 0.05) in *RAD21*-KD versus control OVCAR8 cells. (**G**) qRT-PCR validation of representative ISGs in *RAD21*-KD and control OVCAR8 cells. Data are shown as mean ± SD (*n* = 3, 1-way ANOVA). (**H**) Venn diagram showing the overlaps of RAD21 direct targets and H3K27ac targets. Two clusters (cluster I [common] and cluster II [RAD21 unique]) were divided according to H3K27ac signals. (**I**) Heatmap showing the binding patterns for RAD21 and H3K27ac at accessible regions of cluster I and cluster II genes. (**J**) ChIP-qPCR validation of representative ISGs in *RAD21*-KD and control OVCAR8 cells using antibodies against H3K27ac (left) and RAD21 (right). Data are shown as mean ± SD (*n* = 3, 2-tailed *t* test). (**K**) DNA motif analysis in RAD21 ChIP-Seq peaks showing the significant enrichment of BORIS and TEAD4 motif (hypergeometric test). *P < 0.05; **P < 0.01; ***P < 0.001.

RAD21 is one of the core subunits of the cohesin complex, which plays an important role in gene transcriptional regulation through its associated complexes. To investigate the potential targets of RAD21-dependent gene regulation, we performed RNA sequencing (RNA-Seq) analysis in *RAD21*-depleted OVCAR8 cells. Clustering of 906 differentially expressed genes (DEGs) (*P* < 0.05, |log_2_foldchange| > 1) showed that the majority of DEGs were significantly upregulated upon loss of RAD21, suggesting that RAD21 may act as a repressor to suppress gene expression (Figure 2A). To identify critical and direct RAD21 targets, we performed chromatin immunoprecipitation sequencing (ChIP-Seq) analysis for RAD21 in OVCAR8 cells. ChIP-Seq profiling identified a total of 123,528 RAD21-binding peaks, approximately 67% of them distributed toward intergenic or intronic regions (Figure 2B). Integrative analysis of transcriptome and ChIP-Seq data revealed that 764 genes were potential direct targets of RAD21 (Figure 2C and Supplemental Table 2). Notably, gene set enrichment analysis (GSEA) showed enrichment of upregulated genes in immune-related pathways, including cytokine signaling, IFN signaling, and interleukin signaling (Figure 2, D and E, and Supplemental Figure 2A). Among these immune-regulated genes, IFN-stimulated genes (ISGs) showed the most pronounced changes by RAD21 loss (Figure 2F). Using quantitative reverse transcription PCR (qRT-PCR) analysis, we further confirmed that *RAD21* knockdown mediated by siRNA increased the expression of a subset of ISGs such as *ISG15*, *ISG20*, *IFI44*, and *DDX58* (Figure 2G), which was further confirmed by shRNA in OVCAR8, OVCAR5, and HEY cells (Supplemental Figure 2B). These findings indicated that RAD21 inhibits ISG expression by acting as a repressor.

Given that RAD21 binding sites were identified within a distant enhancer element and organized chromatin loops, we next provided a comparative functional annotation of H3K27ac binding regions by ChIP-Seq (Supplemental Figure 2C). By comprehensive analysis of the transcriptome and chromatin landscape for RAD21 and H3K27ac, we found that 764 RAD21 targets were divided into 2 clusters (cluster I: common binding sites; cluster II: unique binding sites for RAD21) according to H3K27ac signal (Figure 2, H and I). Interestingly, cluster II contained most RAD21-supressed ISGs, suggesting that suppression of these ISGs might be associated with absence of active histone marks (H3K27ac) at these regions. To further evaluate the dynamic changes of RAD21 and H3K27ac peaks in the regions of ISGs, we performed ChIP–quantitative PCR (ChIP-qPCR) in OVCAR8, OVCAR5, and HEY cells with *RAD21* knockdown by shRNA. Indeed, we found a significantly increased co-occupancy of H3K27ac followed by decreased RAD21 (Figure 2J and Supplemental Figure 2, D and E), supporting that loss of H3K27ac is associated with the suppression of RAD21-repressed ISGs. Motif analysis of RAD21 peaks in cluster II using HOMER revealed that BORIS (CTCFL) and CTCF motif were the top hits among the peaks, which were known cohesin interactors (Figure 2K). Notably, the TEAD family were significantly enriched at RAD21 binding regions (Figure 2K). Expression profiling analysis of TEAD1–4 using Gene Expression Profiling Interactive Analysis (GEPIA; http://gepia.cancer-pku.cn/) indicated that only TEAD4 was significantly upregulated in OV (Supplemental Figure 2F). TEAD4, a well-studied transcriptional regulator of stem cell function and tumorigenesis (32, 33), usually forms a complex with YAP to regulate gene expression in OV. These findings suggested that RAD21 may repress ISG expression by attenuating H3K27ac signal, partially through the YAP/TEAD4 transcriptional corepressor complex.

### RAD21 directly interacts with the YAP/TEAD4 transcriptional corepressor complex to suppress ISGs.

**Figure 3:**
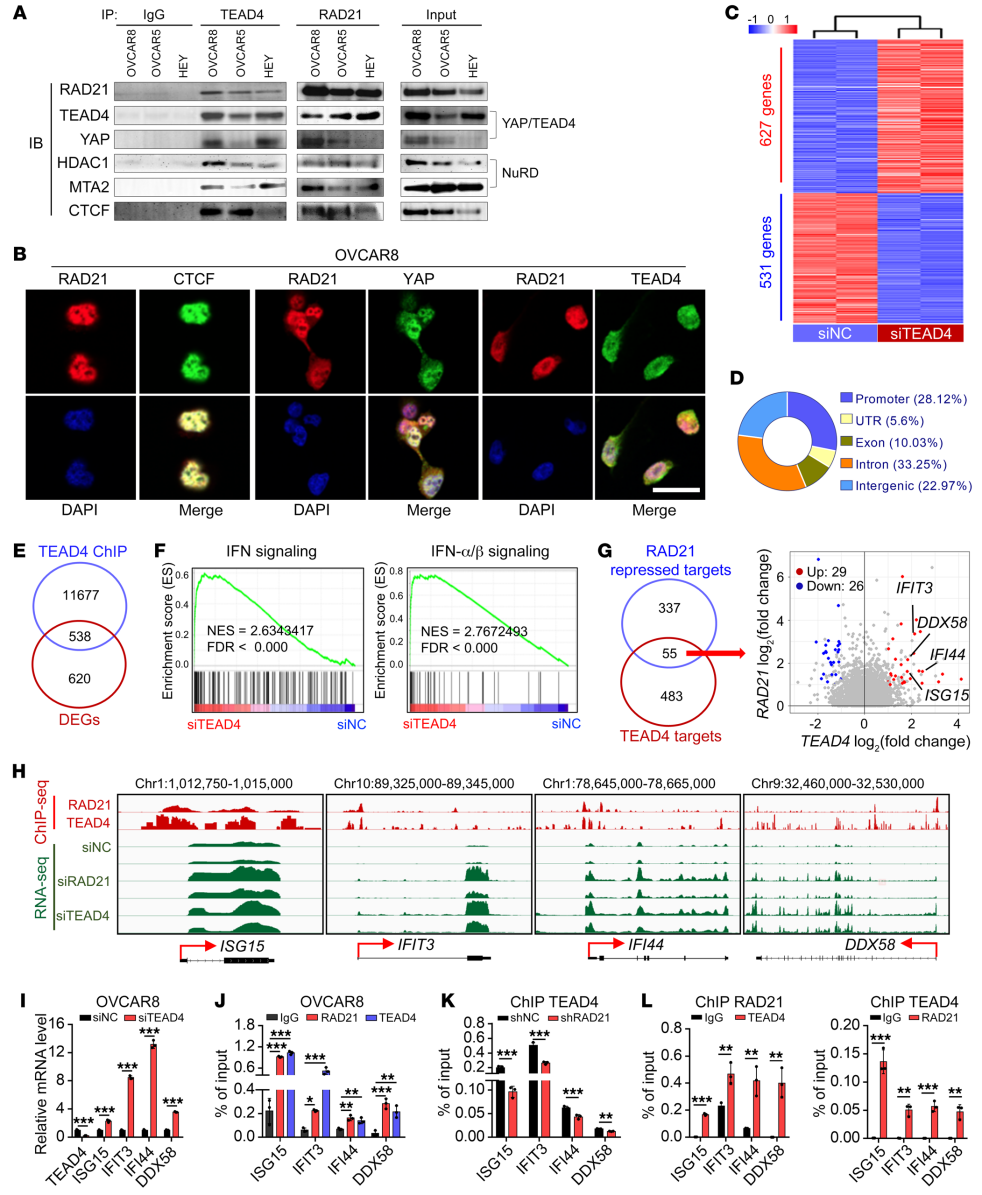
RAD21 directly interacts with YAP/TEAD4 transcriptional corepressor complex to coordinately suppress ISGs. (**A**) Coimmunoprecipitation by anti-IgG, anti-TEAD4, and anti-RAD21 antibodies followed by immunoblotting (IB) with antibodies against the indicated proteins using cell extracts from OVCAR8, OVCAR5, and HEY cells. (**B**) Colocalization of RAD21 with CTCF (left, positive control), YAP (middle), and TEAD4 (right) was visualized by immunofluorescence. Scale bar: 20 μm. (**C**) Heatmap for DEGs (*P* < 0.01, |log_2_foldchange| > 1) in *TEAD4*-KD and control OVCAR8 cells (duplicates). (**D**) Genomic distribution of TEAD4 peaks in OVCAR8 cells. (**E**) Venn diagram showing the overlaps of DEGs and TEAD4 direct targets obtained from TEAD4 ChIP-Seq results. (**F**) GSEA analysis showing that the IFN Signaling and IFN-α/β Signaling pathways were enriched in *TEAD4*-KD cells compared with control cells. (**G**) Venn diagram (left) and scatterplot (right) showing the precise targets repressed by RAD21 and TEAD4. (**H**) Genome browser tracks of RAD21 and TEAD4 ChIP-Seq and RNA-Seq at genomic loci of *ISG15*, *IFIT3*, *IFI44*, and *DDX58*. (**I**) qRT-PCR validation of representative ISGs in *TAED4*-KD and control OVCAR8 cells. (**J**) ChIP-qPCR analysis of RAD21 and TEAD4 occupancy at genomic loci of ISGs *ISG15*, *IFIT3*, *IFI44*, and *DDX58* in OVCAR8 cells. Data are shown as mean ± SD (*n* = 3, 1-way ANOVA). (**K**) ChIP-qPCR analysis of TEAD4 occupancy at genomic loci of ISGs *ISG15*, *IFIT3*, *IFI44*, and *DDX58* in *RAD21*-KD and control OVCAR8 cells mediated by shRNA. (**L**) Re-ChIP analysis showing the concurrent presence of both RAD21 and TEAD4 at genomic loci of ISGs *ISG15*, *IFIT3*, *IFI44*, and *DDX58*. Data in **I**, **K**, and **L** are shown as mean ± SD (*n* = 3, 2-tailed *t* test). *P < 0.05; **P < 0.01; ***P < 0.001.

RAD21 represses ISGs by decreasing the H3K27ac level at specific sites, strongly suggesting the involvement of the nucleosome remodeling and deacetylase (NuRD) complex in the process, which has been proposed to suppress gene expression through the YAP/TEAD4 complex (34). We next investigated whether RAD21 directly interacts with YAP/TEAD4 to form a corepressor complex to suppress gene expression. Using a coimmunoprecipitation assay, we showed a robust interaction in 293T cells cotransfected with exogenous RAD21 and FLAG-tagged TEAD4 (Supplemental Figure 3A). We further confirmed that RAD21 physically interacts with YAP/TEAD4 as well as the crucial NuRD components HDAC1 and MTA2 in three HGSOC cell lines (Figure 3A). In addition, using confocal immunofluorescence, we found a colocalization of RAD21 and YAP/TEAD4 in the nucleus in OVCAR8 cells (Figure 3B). These data raise the possibility that RAD21 forms a corepressor with YAP/TEAD4 and the NuRD complex to suppress ISG expression.

Given the significant colocalization at RAD21 binding regions and physical interaction between RAD21 and YAP/TEAD4 transcriptional corepressor complex, we next investigated potential YAP/TEAD4 targets that are co-suppressed by RAD21. To test the hypothesis, we first performed comparative transcriptome analysis of the TEAD4-regulated genes in OVCAR8 cells with *TEAD4* knockdown, and 1,158 DEGs (*P* < 0.05, |log_2_foldchange| > 1) were identified (Figure 3C). To further identify the direct targets of TEAD4, we performed ChIP-Seq for TEAD4 in OVCAR8 cells. Distributions of TEAD4 peaks across the genome showed that approximately 56.22% of peaks were located at intergenic or intronic regions and 28.12% of peaks located at the promoter (Figure 3D). A total of 538 genes were identified as potential direct targets of TEAD4 through an integrative analysis of transcriptome and TEAD4 ChIP-Seq data (Figure 3E). GSEA showed that the enrichment of upregulated genes were similar with those pathways enriched in RAD21 targets that ranked top pathways were IFN-α/β signaling (Figure 3F), strongly supporting that RAD21 may coordinate with the YAP/TEAD4 complex to repress ISG expression. We next conducted an integrative analysis to explore the precise targets of TEAD4 and RAD21. The results indicated that 55 of 392 RAD21-repressed targets were directly regulated by TEAD4 (Figure 3G). Among these 55 genes, 29 were significantly increased upon silencing of *TEAD4* or *RAD21*, including *ISG15*, *IFIT3*, *IFI44*, and *DDX58*, of which were bound with both RAD21 and TEAD4 (Figure 3H). These findings were further validated by qRT-PCR in OVCAR8, OVCAR5, and HEY cells treated with *TEAD4* siRNA versus scramble control (Figure 3I and Supplemental Figure 3B). Similar results were found in qRT-PCR analysis in OVCAR8, OVCAR5, and HEY cells treated with *YAP* siRNA or YAP inhibitors (Supplemental Figure 3, C and D).

To further investigate how RAD21 coordinates with the YAP/TEAD4 complex to suppress the ISGs, we profiled the binding patterns using TEAD4 ChIP-Seq data and revealed a significant TEAD4 and RAD21 enrichment at the transcription start site (TSS) of these 55 genes (Supplemental Figure 3E). Analysis of a public data set for TEAD4 ChIP-Seq in H1-hESC cell lines from the Encyclopedia of DNA Elements project (ENCODE, GSM1010845, duplicates) further substantiated our findings (Supplemental Figure 3F). Through ChIP-qPCR analysis using RAD21 or TEAD4 antibodies, we demonstrated that RAD21 and TEAD4 co-occupied in TSS of selected ISGs, whereas TEAD4 enrichment was reduced upon *RAD21* knockdown (Figure 3, J and K). In agreement with ChIP-qPCR, sequential ChIP (Re-ChIP) experiments further confirmed that RAD21 and TEAD4 bound to the same genomic sites at these ISGs (Figure 3L). Together, these findings revealed that overexpressed *RAD21* suppressed the IFN signaling genes through YAP/TEAD4 repressive complex.

### RAD21 inversely correlates with IFN signaling activity in OV.

**Figure 4:**
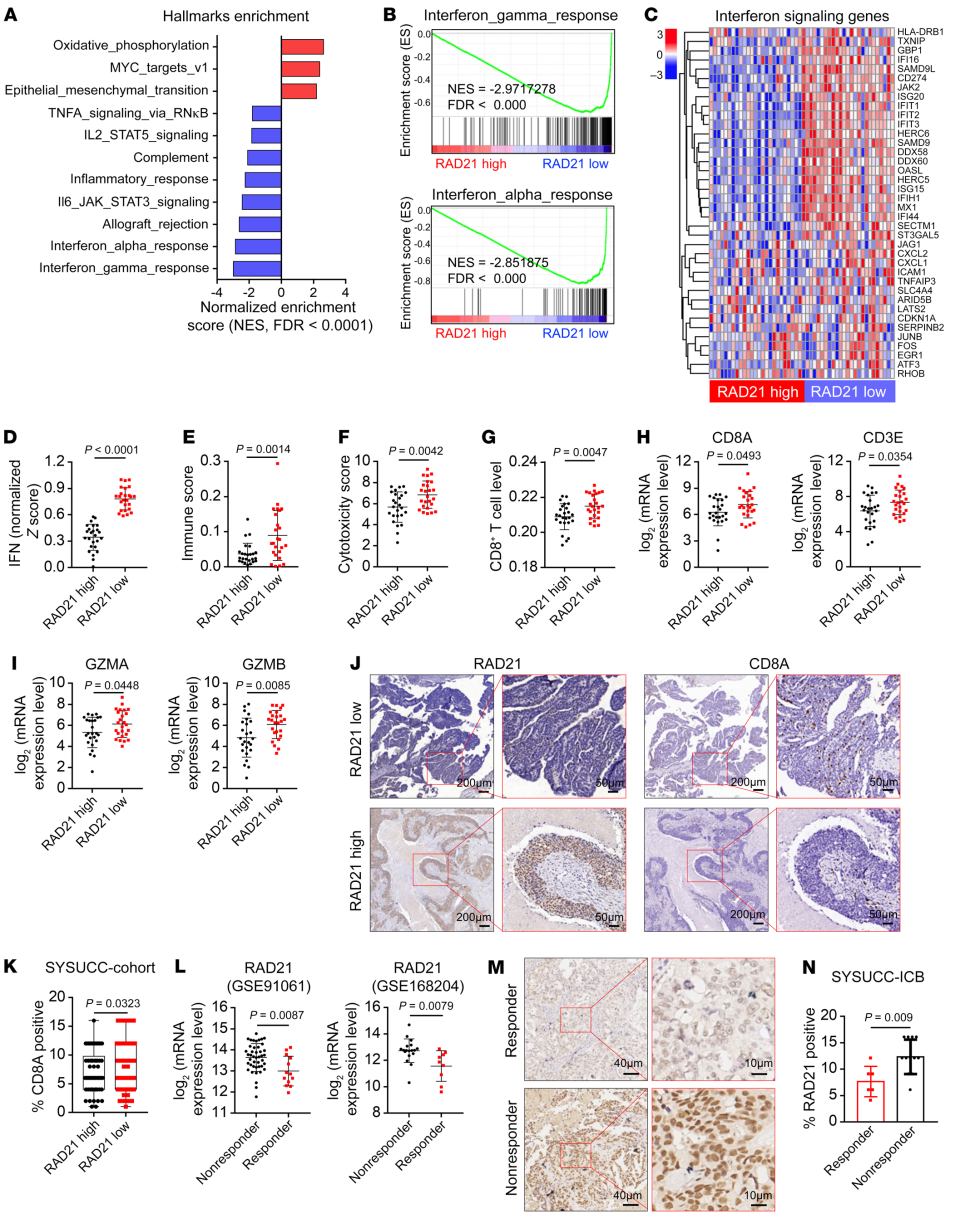
RAD21 inversely correlates with IFN signaling activity in OV. (**A**) Normalized enrichment scores correlated with RAD21 expression using Hallmark gene sets from TCGA-OV database (high vs. low RAD21 expression, top vs. bottom 10%; *n* = 25 per group). (**B** and **C**) GSEA analysis (**B**) and heatmaps (**C**) showing the inverse correlation between RAD21 expression and the IFN signaling pathways and genes in the TCGA-OV database. (**D**–**I**) Comparative analysis showing the association of high expression of RAD21 with low expression of IFN activity (**D**), immune score (**E**), cytotoxicity score (**F**), infiltration levels of CD8^+^ T cells (**G**), and expression of T cell marker genes *CD8A*, *CD3E* (**H**), *GZMA*, and *GZMB* (**I**) in patients with OV from the TCGA database. (**J** and **K**) Representative IHC staining (**J**) and quantification (**K**) showing the inverse correlation between RAD21 expression and CD8A expression in ovarian tumors (SYSUCC cohort). (**L**) Correlation of RAD21 mRNA levels with response to ICB in patients with melanoma treated with PD-1, PD-L1, or CTLA4 mAbs (GSE91061 and GSE168204). (**M** and **N**) Representative IHC images (**M**) and quantification (**N**) for RAD21 expression in OV responders (*n* = 6) versus nonresponders (*n* = 12) to immune checkpoint inhibitors. Data in **D**–**I**, **K**, **L**, and **N** are shown as mean ± SD (2-tailed *t* test).

Amplified *RAD21* suppresses ISG gene expression in OV cell lines, suggesting that *RAD21* expression may negatively correlate with IFN activity. We next interrogated public transcriptome data and CNA data for pan-cancer analysis in the TCGA database. The results demonstrated that *RAD21* was amplified and overexpressed in various human tumors, including OV and skin cutaneous melanoma (SKCM) (Supplemental Figure 4A). *RAD21* expression was inversely correlated with canonical immune signatures such as IFN-α/γ response signaling (Figure 4A). GSEA analysis also revealed a negative correlation between expression of *RAD21* and IFN-α/γ response signaling in OV (Figure 4B). In addition, *RAD21* expression level was negatively correlated with the majority of ISGs that were identified as direct targets of RAD21 and TEAD4 (Figure 4C), a relationship that was also observed in an analysis of RNA-Seq data from 1,377 human cancer cell lines (Cancer Cell Line Encyclopedia) (Supplemental Figure 4B). By analyzing IFN response activity (IFN score) as previously described (35, 36), we showed an anti-correlation between *RAD21* expression level and IFN score (Figure 4D), further supporting that RAD21 may suppress IFN response signaling. We next defined the connection between RAD21 and immune microenvironment by using TIMER 2.0 (http://timer.cistrome.org) (37). The results showed that patients with low *RAD21* levels exhibited higher immune scores and cytotoxicity scores and significantly increased infiltration levels of CD8^+^ T cells (Figure 4, E–G). We further showed that tumors with lower *RAD21* levels expressed higher T cell markers such as *CD8A*, *CD3E*, *GZMB*, and *GZMA* (Figure 4, H and I). To further verify these results, we assessed RAD21 and CD8A expression with immunohistochemistry staining in the same cohorts of HGSOC tissues. The intensity of CD8A expression was lower in HGSOC cases with higher RAD21 levels (Figure 4, J and K). In addition, we found that the *RAD21* level was overexpressed and inversely correlated with IFN signaling in patients with SKCM in the TCGA database (Supplemental Figure 4, C–I).

Since suppression of IFN signaling pathways is associated with resistance to immunotherapy (36), we next examined the clinical relevance of RAD21 in immunotherapy. We evaluated *RAD21* expression in 2 independent clinical immune checkpoint blockade (ICB) cohorts with advanced melanoma (GSE91061 and GSE168204). The results indicated that the expression of *RAD21* was significantly lower in ICB responders compared with nonresponders (Figure 4L). In addition, the IFN response activity was inversely associated with *RAD21* level in these 2 cohorts (Supplemental Figure 4J). Correlation analysis showed that RAD21 expression level was negatively correlated with IFN activity in responders (Supplemental Figure 4K). To further confirm the role of RAD21 in OV patients who were treated with anti–PD-1 immunotherapy, we detected the expression of RAD21 in 18 OV biopsies by IHC analysis. The data showed that RAD21 expression was significantly higher in nonresponders than in responders (*P* = 0.009) (Figure 4, M and N), indicating that RAD21 overexpression was associated with inferior response to ICB in OV. Collectively, these findings demonstrated that *RAD21* was inversely correlated with IFN signaling activity, suggesting that *RAD21* overexpression may confer immune resistance to ICB through suppression of IFN activity.

### RAD21 ablation induces T cell activation in vitro.

**Figure 5:**
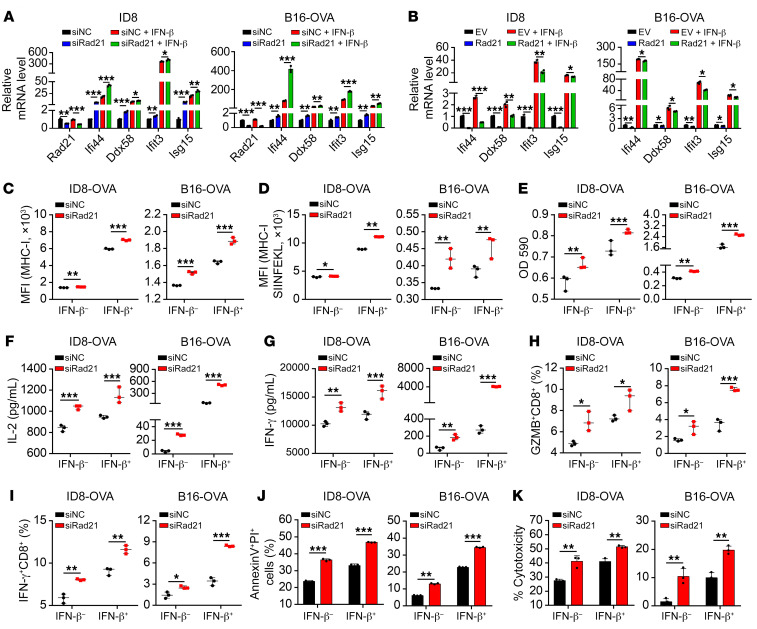
RAD21 ablation induces T cell activation in vitro. (**A** and **B**) qRT-PCR validation of representative ISGs *Ifi44*, *Ddx58*, *Ifit3*, and *Isg15* in *Rad21*-KD or *Rad21*-overexpressed and control ID8 cells and B16-OVA cells in the presence or absence of IFN-β treatment. (**C** and **D**) Expression levels of MHC-I and MHC-I–SIINFEKL on *Rad21*-KD and control ID8-OVA and B16-OVA cells in the presence or absence of IFN-β treatment were determined by FACS. (**E**–**I**) *Rad21*-KD and control ID8-OVA and B16-OVA cells were treated with vehicle or IFN-β and then cocultured with B3Z cells or OT-I cells, after which B3Z activation was determined by LacZ activity (**E**) and OT-I activation was determined by secretion of IL-2 (**F**) and IFN-γ (**G**) and expression of effector molecules GZMB (**H**) and IFN-γ (**I**). (**J** and **K**) The cytotoxic effect of OT-I was measured by annexin V/propidium iodide staining (**J**) and LDH release (**K**) of ID8-OVA and B16-OVA cells after coculture with OT-I for 48 hours. Data in **A**–**K** are shown as mean ± SD (*n* = 3, 2-tailed *t* test). *P < 0.05; **P < 0.01; ***P < 0.001.

IFN signaling has been correlated with response to immunotherapy (36, 38), which prompted us to hypothesize that RAD21 may promote immune evasion. To investigate the role of RAD21 in tumor immunity, we selected both murine ID8 ovarian cancer cells and mouse OVA-expressing B16 melanoma cells (B16-OVA), two well-established mouse synergic tumor models with a higher expression of Rad21 (Supplemental Figure 5A). We next generated *Rad21*-knockdown cells with specific siRNA in ID8 and B16-OVA cells and treated them with recombinant IFN-β. Consistent with the findings in human cancer cell lines, the canonical IFN signaling genes such as *Isg15*, *Ifi44*, *Ifit3*, and *Ddx58* were induced at a significantly higher magnitude in *Rad21*-knockdown cells upon IFN-β stimulation (Figure 5A). Conversely, ectopic expression of RAD21 suppressed the expression of ISGs and attenuated the response to IFN-β stimulation (Figure 5B and Supplemental Figure 5, B and C). MHC-I expressed on the cell surface is one of the key tumor antigen presentation machinery components and is essential for T cell recognition and killing (39). We detected a higher level of MHC-I in *Rad21*-depleted ID8-OVA cells or B16-OVA compared with scramble control cells (Figure 5C), as well as increased MHC-I–bound SIINFEKL (OVA epitope peptide) complex expression in Rad21-depleted cells (Figure 5D). In line with the siRNA effect, we further confirmed our findings by using CRISPR/Cas9–mediated gene knockout with 2 individual sgRNAs targeting *Rad21* (Supplemental Figure 5, D and E).

Since IFN signaling activity is positively correlated with T cell activation (38), we next determined whether *RAD21* was associated with T cell activation. We adopted an *IL-2* promoter–driven LacZ assay, which reflected T cell–intrinsic IL-2 expression in a specific coculture system using ID8-OVA or B16-OVA and OVA-specific CD8^+^ T cell hybridoma B3Z cells (40). The absorbance at 590 nm manifested that *RAD21* depletion significantly enhanced *IL-2* promoter activity (Figure 5E and Supplemental Figure 5F). Consistently, the supernatant levels of the T cell–derived cytokines IL-2 and IFN-γ were dramatically increased in primary OT-I T cells when cocultured with ID8-OVA or B16-OVA cells treated with siRNA or sgRNAs versus scramble control (Figure 5, F and G, and Supplemental Figure 5G). In addition, the proportion of CD8^+^ T cells expressing the effector molecules IFN-γ and granzyme B (GZMB) also increased in *Rad21* siRNA–carrying cells after coculture (Figure 5, H and I). We next examined the cytotoxic killing effect of OT-I cells on B16-OVA and ID8-OVA cells in a coculture system. Cell death was detected by annexin V–FITC apoptosis assay, while cytotoxicity was determined by lactate dehydrogenase (LDH) release assay. After coculture with OT-I T cells, the proportion of apoptotic cells and LDH release was significantly increased at a higher magnitude in *Rad21*-knockdown cells upon IFN-β stimulation in both B16-OVA and ID8-OVA (Figure 5, J and K). Together, these findings demonstrated that overexpression of RAD21 in tumor cells suppressed T cell activation and cytotoxic T cell activity.

### RAD21 suppresses antitumor immunity in vivo.

**Figure 6:**
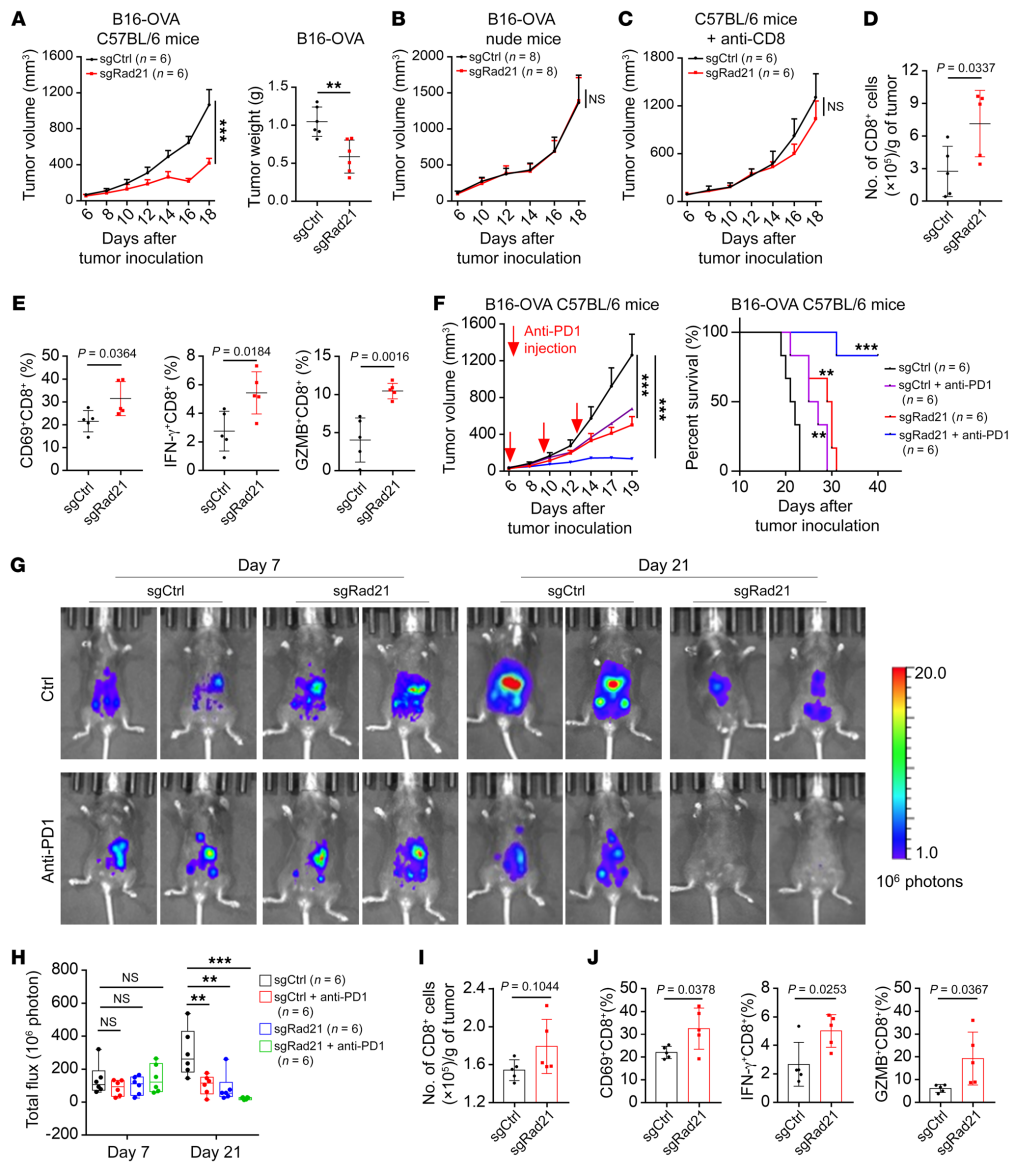
RAD21 suppresses antitumor immunity in vivo. (**A**) Tumor volume and tumor weight over time in C57BL/6 mice implanted with *Rad21*-KO and control B16-OVA mouse cells. Data are shown as mean ± SEM (2-way ANOVA) for tumor volume and as mean ± SD (2-tailed *t* test) for tumor weight (*n* = 6 mice per group). (**B** and **C**) Tumor volume in nude mice (*n* = 8 mice per group) (**B**) and C57BL/6 mice (*n* = 6 mice per group) (**C**) implanted with *Rad21*-KO and control B16-OVA mouse cells. Mice were pretreated with CD8-depleting antibodies at –1, 2, and 5 days. Data are shown as mean ± SEM (2-way ANOVA). (**D** and **E**) Flow cytometry analysis showing the numbers of tumor-infiltrating CD8^+^ T cells (**D**) and expression of activation marker CD69 and effector molecules IFN-γ and GZMB (**E**) in CD8^+^ T cells. Data are shown as mean ± SD (*n* = 5, 2-tailed *t* test). (**F**) Mice with established *Rad21*-KO and control B16-OVA tumors were treated with anti–PD-1 at indicated time points. Tumor volume and survival rates are shown. Data are shown as mean ± SEM (2-way ANOVA). (**G**) Representative bioluminescence images of mice with established *Rad21*-KO and control ID8 tumors treated with anti–PD-1 formed by intraperitoneal injection at day 9 and day 12. (**H**) The bar graph shows the change in bioluminescence in mice. Data are shown as mean ± SEM (1-way ANOVA). (**I** and **J**) Flow cytometry analysis showing the numbers of tumor-infiltrating CD8^+^ T cells (**I**) and expression of CD69 and effector molecules IFN-γ and GZMB (**J**). Data are shown as mean ± SD (2-tailed *t* test). **P < 0.01; ***P < 0.001.

Our in vitro studies demonstrated that RAD21 inhibits IFN signaling genes and T cell activation, suggesting that RAD21 may play a role in antitumor immunity. To validate these findings in vivo, we inoculated B16-OVA cells expressing sgRad21 and scramble control in a mouse syngeneic tumor model. The results showed that Rad21 depletion significantly suppressed tumor growth in immune-competent C57BL/6 mice bearing B16-OVA tumor (Figure 6A). Intriguingly, there were no significant differences in tumor growth or tumor weight derived from the same type of tumor cells when inoculated on T cell–deficient nude mice (Figure 6B and Supplemental Figure 6A). In addition, sgRad21-mediated B16-OVA tumor inhibition was abrogated by pretreatment of anti-CD8 depletion antibody, further suggesting that CD8^+^ T cells are required for the retarded tumor growth of sgRad21-expressing tumors (Figure 6C and Supplemental Figure 6B). Consistently, we found that levels of tumor-infiltrating CD8^+^ T cells were significantly increased in mice bearing sgRad21 B16-OVA tumors by using flow cytometry analysis (Figure 6D). The levels of T cell activation markers CD69, IFN-γ, and GZMB in tumor-infiltrating CD8^+^ T cells were increased in sgRad21 B16-OVA tumors compared with scramble sgRNA–carrying tumors (Figure 6E). Furthermore, IHC analysis confirmed that Rad21 ablation enhanced the level of tumor-infiltrating CD8^+^ T cells (Supplemental Figure 6, C and D). Notably, RAD21 depletion significantly increased PD-L1 expression in both human and murine cancer cells (Supplemental Figure 6, E–I). These observations suggested a potential antitumor effect of Rad21 inhibition in anti–PD-1 immunotherapy.

We next examined whether RAD21 affects the therapeutic efficacy of immunotherapy. We treated mice bearing sgRad21 or scramble B16-OVA tumors with anti–PD-1 mAb. The results indicated that sgRad21 treatment significantly enhanced the antitumor efficacy of anti–PD-1 as shown by slower tumor growth and higher survival rate (Figure 6F). To further determine whether RAD21 could suppress antitumor immunity in OV, we established an isogenic ID8 cell line with sgRNA-mediated Trp53 knockout, which could mimic several key aspects of HGSOC (41). Consistently, Trp53 depletion significantly promoted cell proliferation and colony formation (Supplemental Figure 6, J and K). We next conducted Rad21 knockout with sgRNA in ID8^Trp53–/–^ cells and detected a higher level of MHC-I in *Rad21*-KO cells than in scramble control cells (Supplemental Figure 6L). We engrafted ID8^Trp53–/–^ cells expressing sgRad21 and scramble control in a syngeneic mouse model by intraperitoneal injection and treated the mice with anti–PD-1 mAb. Compared with control tumors, *Rad21*-KO tumors showed a remarkable response to anti–PD-1 antibody (Figure 6, G and H). The numbers and the levels of CD69, IFN-γ, and GZMB in tumor-infiltrating CD8^+^ T cells were increased in sgRad21 ID8^Trp53–/–^ tumors compared with scramble sgRNA–carrying tumors (Figure 6, I and J). These data were concordant with B16-OVA tumors and strengthened our findings that RAD21 amplification could promote immune evasion and attenuate the tumor response to anti–PD-1 immunotherapy.

## Discussion

Resistance to platinum-based chemotherapy and limited clinical responses to immunotherapy remain two major unsolved problems in HGSOC (4, 42), and novel approaches to overcome them are urgently required. Recently, amplification of *CCNE1* or *PRKCI* has been reported to promote HGSOC, by enhancing an immunosuppressive tumor microenvironment in the case of *PRKCI* amplification (7), conferring chemoresistance in OV (43). Tumor immune evasion is associated with immunotherapy failure and chemoresistance (44). Therefore, the systematic genome-wide examination of CNAs, a key genetic feature of HGSOC recognized to promote chemoresistance and immune suppression, could have a major impact on improving clinical outcomes.

In this study, we systematically investigated potential driver genes in HGSOC that are regulated by CNAs and examined their roles in immune evasion. We identified RAD21 amplification as a major oncogenic driver event that correlates with poor prognosis in HGSOC. Our results indicate that amplified RAD21 forms a transcriptional corepressor complex to suppress its downstream targets by reducing the active histone marker H3K27ac. This is consistent with previous studies reporting that RAD21 coordinates with transcriptional corepressors or coactivators to form cohesin complexes, which repress or activate the downstream targets (17, 18, 22). We integrated our transcriptome and RAD21 ChIP-Seq profiling analysis and revealed that upon loss of RAD21, IFN signaling pathways were significantly enriched and upregulated, suggesting a potential role of RAD21 in antitumor immunity in HGSOC.

YAP and TEAD4 are potent oncogenic transcriptional coactivators implicated in multiple processes, including tumorigenesis, chemoresistance, and immune evasion (7, 45, 46). However, the roles of YAP/TEAD4 as a transcriptional corepressor to suppress the expression of ISGs remain unclear. In this study, we demonstrated that RAD21 recruits YAP/TEAD4 and NuRD corepressor complex to attenuate H3K27ac levels on gene loci and impedes expression of ISGs. This critical role of the RAD21–YAP/TEAD4 complex in HGSOC tumor biology hints at its potential to be targeted therapeutically in HGSOC.

**Figure 7:**
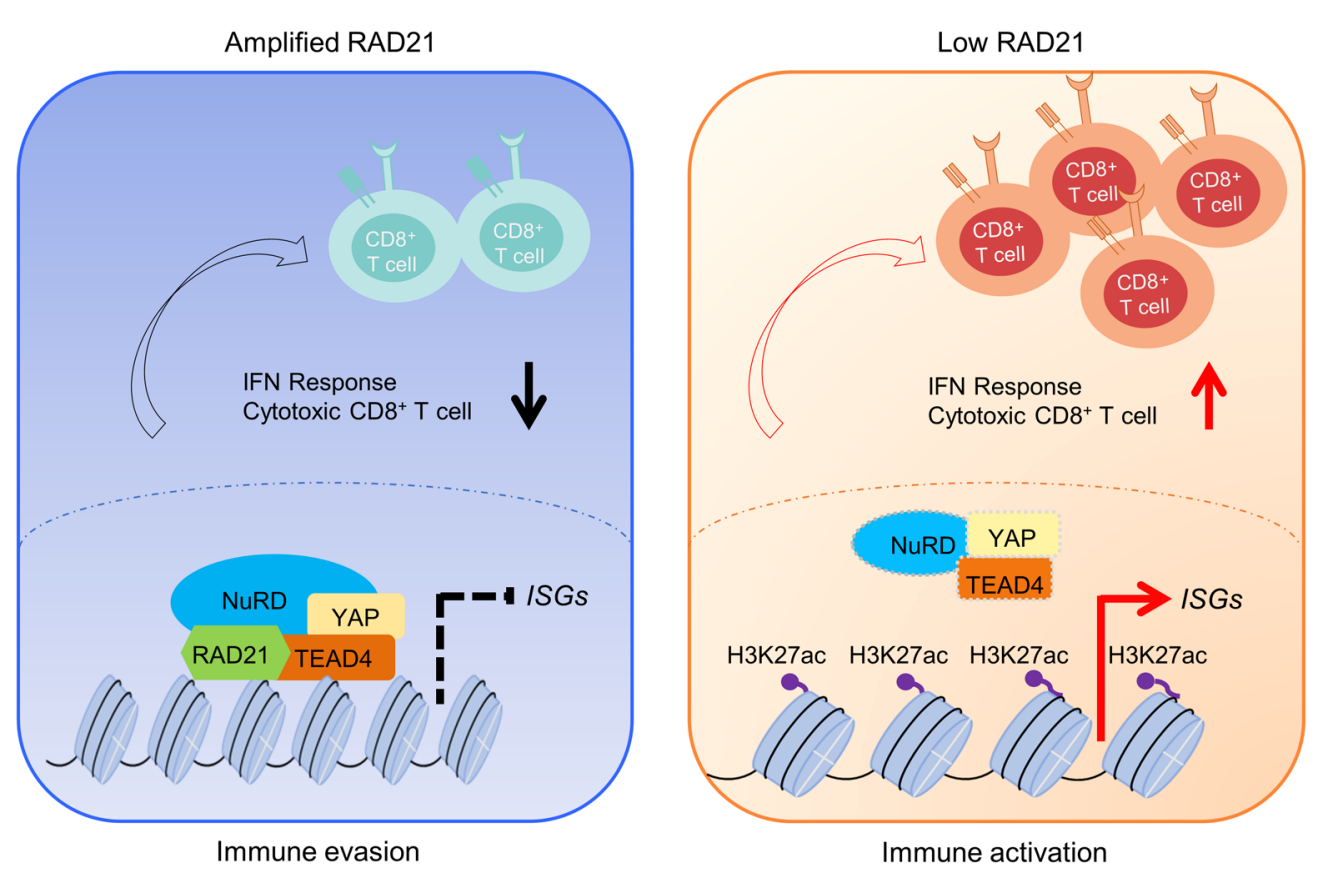
Schematic model for the role of RAD21 in modulating antitumor immunity in OV. Left: Amplification of RAD21 recruits YAP/TEAD4 and NuRD corepressor complex to suppress ISG expression, which contributes to immune evasion. Right: RAD21 ablation reactivates IFN signaling to enhance antitumor immunity in OV.

Despite the remarkable clinical successes of immune checkpoint blockade (ICB) therapeutics, the benefit to the majority of patients with OV is limited (4). The key determinants mediating response to ICB remain elusive, highlighting the urgent need to investigate the biology underpinning immune evasion in OV. Multiple factors may influence the ICB response, including, but not limited to, the heterogeneity of the complex tumor microenvironment, such as tumor immune phenotypes or tumor-infiltrating lymphocyte patterns (47); tumor PD-L1 expression (48); and other “don’t eat me” signals like CD24 (49). Increasing evidence suggests that expression of IFN signaling genes in cancer cells is required for ICB response (50, 51). Activation of IFN signaling pathways is essential for antigen presentation and T cell recognition and killing, as well as immunotherapy effect (52). Recent studies demonstrated that loss of IFN signals or antigen presentation mediated by FOXA1 and ADAR1 is associated with poor antitumor immunity (36, 53). In addition, chromosomal aneuploidy with copy number amplifications could suppress immune surveillance by decreasing cytotoxic infiltrating immune cells, especially CD8^+^ T cells (54). Inhibition of G9a/DNA methyltransferase activity combined with anti–PD-L1 therapy significantly improved the antitumor effect (55). Lung cancers with MET amplifications were resistant to ICB therapy through decreasing STING levels and antitumor T cell infiltration (56). A recent study has shown that CTCF and the cohesin complex proteins were negative regulators of ISGs and PD-L1 expression, which depends on the activity of cohesin complex and NF-κB expression signatures (57). Loss of any subunits of cohesin complexes or STAG2 mutations induced NF-κB activity and JAK-STAT signaling. In our study, we demonstrated that amplified RAD21 could interact with YAP/TEAD4 corepressor to suppress expression of IFN-inducible genes through chromatin remodeling. Disruption of this corepressor complex restored IFN signaling by increasing chromatin accessibility (Figure 7). We further showed that RAD21 depletion elevated antigen presentation, T cell activation, and antitumor efficacy of anti–PD-1 therapy in vitro and in vivo, supporting the potential of RAD21 targeting to exert profound effects on ICB response. Previous studies have demonstrated a role of RAD21 amplification in chemoresistance (25, 58). Our study provides a new mechanistic insight into regulation of immune response by RAD21 and indicates that RAD21 amplification can contribute to disease progression and drug/therapeutic resistance phenotype through multiple mechanisms, which warrant further investigation.

In summary, this study provides key evidence that amplification of RAD21, a major oncogenic driver event in HGSOC, is a potential prognostic indicator of poor survival outcomes. We provide initial evidence for a unique regulatory complex, RAD21–YAP/TEAD4, that epigenetically silences IFN signaling genes. Furthermore, we verified a role of RAD21 in suppressing cancer immune response, which engenders an immunosuppressive tumor microenvironment with poor infiltration of cytotoxic T cells. RAD21 blockade improves response to anti–PD-1 therapy and suppresses tumor progression. Despite advances in the genetic and molecular characterization of OV, few discoveries have been successfully translated into biomarkers for routine clinical use or led to tangible improvements in cancer therapy. Our study sheds new light on the regulatory mechanisms of RAD21 in immune evasion, providing grounds for the immune therapeutic targeting of cohesin complexes in HGSOC clinically.

## Methods

### Cell lines and reagents.

A2780 and COV504 cells were purchased from the European Collection of Cell Cultures (Salisbury, UK). OVCAR8 cells were obtained from the National Cancer Institute (USA), KURAMOCHI cells were obtained from the Japanese Collection of Research Bioresources Cell Bank (Tokyo, Japan), and DOV13 cells were purchased from BioVector (BioVector NTCC). All other human cell lines were from the American Type Culture Collection. Short Tandem Repeat (STR) authentication of cell lines was done by the authors. Mouse ovarian carcinoma cell line ID8 was purchased from the Cell Bank of Chinese Academy of Sciences (Shanghai, China). The B16-OVA cell line (C57BL/6 mouse melanoma) was constructed by overexpression of OVA in B16 cells, and B3Z hybridoma cells were a gift from Nilabh Shastri (University of California, Berkeley, California, USA) (59). Cell lines were cultured in DMEM or RPMI 1640 medium supplemented with 10% FBS, 1% penicillin-streptomycin (Gibco), and maintained at 37°C in a humidified 5% CO_2_ atmosphere and routinely tested negative for mycoplasma. Carboplatin were purchased from Selleck Chemicals. Doxycycline and verteporfin were from TopScience, respectively. Mouse IFN-β was obtained from R&D Systems. Stock solutions were diluted and stored according to the manufacturer’s protocols.

### Colony formation assay.

For colony formation in monolayer culture, 1 × 10^4^ cells were seeded in 6-well plates and maintained for 10–12 days. Colonies were stained with gentian violet after methanol fixation.

### IHC and FISH analysis.

The FFPE sections were dewaxed and rehydrated through graded alcohol to water before antigen unmasking, followed by treatment with 3% hydrogen peroxide. Then slides were blocked using a blocking solution and incubated with optimally diluted antibody targeting RAD21 (Abcam, ab992), human CD8 (ZSGB-BIO, ZA-0508), and mouse CD8 (Cell Signaling Technology [CST], 98941) overnight at 4°C. Detection was carried out using Dako REAL HRP Rabbit/Mouse detection kit for 30 minutes, and the signal was subsequently detected by the chromogenic substrate (Dako). Procedures for the FISH experiment were performed according to the manufacturer’s instructions (Wuhan HealthCare Biotechnology), and FISH images were captured with a Zeiss LSM510 confocal microscope. The optimal cutoff point of RAD21 expression and copies was conducted based on X-tile software (X-tile 3.6.1) (60). The patient cohort from Sun Yat-sen University Cancer Center was designated the “SYSUCC cohort,” and detailed patient characteristics are listed in Supplemental Table 3.

### Immunoblotting and immunoprecipitation analysis.

Immunoblotting analyses were performed as described previously (61). Briefly, protein extracts were prepared with RIPA cell lysis buffer with the protease inhibitor cocktail (Roche) and measured using Pierce BCA protein assay (Thermo Fisher Scientific). Lysates and prestained marker (GenStar) were subjected to SDS-PAGE and transferred to PVDF membrane for immunoblotting analysis. For immunoprecipitation analysis, cells were lysed with IP lysis buffer containing 50 mM Tris-HCl, 150 mM NaCl, 1.0% NP-40, and complete protein inhibitor cocktail for 30 minutes on ice. Cell lysates were precleared and immunoprecipitated with indicated antibodies using protein G Dynabeads (Thermo Fisher Scientific) overnight at 4°C. Immunoprecipitates were washed with IP buffer, then boiled in SDS sample buffer and analyzed by immunoblotting. Anti-RAD21 (Abcam, ab992), anti-TEAD4 (Santa Cruz Biotechnology, sc-101184), anti-IgG (Santa Cruz Biotechnology, sc-2025), and anti-FLAG (Sigma-Aldrich, F1804) were used for immunoprecipitation. The following antibodies were used for immunoblotting: anti-RAD21 (Abcam, ab992), anti-actin (CST, 8456S), anti-YAP (CST, 14074S), anti-TEAD4 (Abcam, ab97460), anti-FLAG (Sigma-Aldrich, F1804), anti-HDAC1 (CST, 34589S), anti-MTA2 (Abcam, ab8106), anti-CTCF (CST, 3418S), and anti-GAPDH (CST, 2118S).

### RNA interference.

Cells were seeded into 6-well plates and transfected with indicated siRNAs using Lipofectamine RNAi-MAX (Life Technologies) according to the manufacturer’s instructions. All siRNAs were obtained from GenePharma, and the sequences are available in Supplemental Table 4.

### Confocal analysis.

For immunofluorescence, cells were fixed with 3.7% paraformaldehyde in PBS and permeabilized with 0.2% Triton X-100. After 2 additional washes, the cells were blocked with 1% BSA for 1 hour at room temperature and subsequently incubated with the indicated primary antibodies (anti-RAD21, anti-YAP, anti-TEAD4, and anti-CTCF) and Alexa Fluor 633–conjugated secondary antibodies (Thermo Fisher Scientific). DAPI was used for nuclear staining for 15 minutes, and the stained cells were examined using a Zeiss LSM510 confocal microscope.

### Plasmid construction and virus infection.

Total RNA was extracted and reversed to cDNA using RNeasy Mini Kit (QIAGEN) and reverse transcription kit (AT341-02, TransGen Biotech) following the manufacturers’ protocols. Human RAD21 and mouse Rad21 were amplified from HEK293T cDNA and ID8 cDNA, respectively, and then cloned into the pCDH-CMV-MCS-EF1-copGFP lentiviral expression vector (System Biosciences). FLAG-tagged human TEAD4 was cloned into pcDNA3.0/FLAG vector (Invitrogen). shRNAs targeting human RAD21 were cloned into PLKO.1 plasmid (Addgene plasmid 10878). For mouse Rad21 and Trp53 gene knockout, sgRNA sequences were designed using the Optimized CRISPR Design (http://chopchop.cbu.uib.no/) and inserted into the LentiCRISPR v2 vector (Addgene plasmid 52961) containing the *Streptococcus pyogenes* Cas9 nuclease gene. The lentiviral vectors were transfected into HEK293T packaging cells with Lipofectamine 2000 (Thermo Fisher Scientific). The viral supernatants were passed through a 0.45 μm nitrocellulose filter and were used to infect target cells. At 48 hours after transfection, stably transfected cells were sorted with GFP by flow cytometry (BD Biosciences) or selected with 1.0 μg/mL puromycin (Sigma-Aldrich) for 4 days. For transient transfection of RAD21 and TEAD4 in HEK293T cells, Lipofectamine 2000 was used following the manufacturers’ protocols. All the sequences of primers are listed in Supplemental Table 4.

### qRT-PCR and RNA-Seq.

Total RNA was extracted from cells using RNeasy Mini Kit (QIAGEN) according to the manufacturer’s protocol. For qRT-PCR analysis, RNAs were reversed to cDNA using a reverse transcription kit (AT341-02, Transgen Biotech) and quantitative kit (N30920, Transgen Biotech) on a Bio-Rad CFX Real-Time PCR machine. For RNA-Seq, the libraries were prepared using TruSeq Stranded RNA HT kit 96 samples Ribo-Zero Gold (Illumina) following the manufacturer’s instructions. RNA sequencing was performed on a NovaSeq sequencer (Illumina). All qPCR primer sequences are listed in Supplemental Table 5.

### ChIP, ChIP-qPCR, and ChIP-Seq.

Cells were cross-linked with 1% formaldehyde and quenched with 0.125 M glycine. The cross-linked cells were lysed (50 mM Tris-HCl [pH 8.0], 10 mM EDTA, 1% SDS) and sonicated on ice using Bioruptor (Diagenode, Belgium). The lysates were precleared and immunoprecipitated with protein G Dynabeads (Invitrogen) and incubated with antibody-beads complex overnight at 4°C. Immunoprecipitations were eluted, reverse cross-linked, and purified for ChIP-DNA. For ChIP-qPCR analysis, the ChIP-DNA was quantitated using SYBRTM Green PCR master mix (Thermo Fisher Scientific) on a Bio-Rad CFX Real-Time PCR machine. The enrichment of specific genomic regions was accounted relative to the input DNA. For ChIP-Seq, library construction and sequencing were performed as described previously (62). We used the following antibodies in the ChIP studies: anti-H3K27ac (Abcam, ab4729), anti-RAD21 (Abcam, ab992), and anti-TEAD4 (Santa Cruz Biotechnology, sc-101184). The ChIP primer sequences are listed in Supplemental Table 6.

### Bioinformatics analyses.

For RNA-Seq analysis, raw reads were cleaned with fastp (version 0.12.5) and then aligned to human reference genome (GRCh38, hg38) using STAR (version 2.7.0f) with default settings (63, 64). Expression level was estimated using RSEM (65). The DESeq2 package was used to normalize the raw counts and identify differentially expressed genes (|log_2_foldchange| > 1 and adjusted *P* value < 0.05). Pathway enrichment was assessed by gene set enrichment analysis (GSEA) desktop software using RECTOME gene sets (66). For ChIP-Seq analysis, raw reads were cleaned with fastp and mapped to the human reference genome (GRCh38, hg38) using Bowtie2 (version 2.3.2) with default settings (67). Peaks were called on each individual sample using MACS2 (version 2.1) with default settings (-q 0.01) (68). Peaks were annotated with ChIPseeker (version 1.14.2) and visualized using deepTools (69, 70). Alignments for individual genes were visualized using the Integrative Genomics Viewer (71) (version 2.4.19). DNA motif analysis across the RAD21 or TEAD4 peaks was carried out using HOMER (homer.salk.edu) (72). Customized R scripts and Adobe Illustrator (2017 CC) were used to generate better visualizations. For TCGA data analysis, the University of California, Santa Cruz, Xena Browser (https://xenabrowser.net) was used to access TCGA OV and SKCM cohorts for gene expression levels; high versus low RAD21 expression was defined as the top 10% versus the bottom 10%.

### Re-ChIP assay.

Cells were cross-linked for 20 minutes at room temperature with 1% formaldehyde and quenched with 0.125 M glycine for 5 minutes. The detailed procedures were performed as previously described (73). Briefly, the first immunoprecipitation was carried out with antibody cross-linked to protein G Dynabeads using dimethyl pimelimidate•2 HCl (DMP; Pierce), and the second immunoprecipitation was performed after ChIP experiments. Anti-TEAD4 (Santa Cruz Biotechnology, sc-101184), anti-IgG (Santa Cruz Biotechnology, sc-2025), and anti-RAD21 (Abcam, ab992) were used for immunoprecipitation.

### LacZ reporter assays and ELISA analysis.

The LacZ activity was measured according to previously described methods (40). In brief, the B16-OVA cells were cocultured with B3Z cells in 96-well plates for 24 hours, then cells were lysed and freeze-thawed at –80°C. A total of 150 μL/well substrate solution (50 μL PBS containing 0.5% BSA plus 100 μL β-galactosidase buffer containing 1 mg/mL chlorophenol red β-d-galactopyranoside) was mixed well and added into each well. The plate was incubated at 37°C for 12–18 hours, and the absorbance at 590 nm was measured using an Infinite M200 plate reader (Tecan). Supernatant levels of IL-2 and IFN-γ were measured by ELISA kits (Invitrogen, 88-7024, 88-7314) following the manufacturer’s instructions.

### T cell activation and FACS analysis.

B16-OVA tumor cells infected with scramble sgRNA or sgRAD21 were stained with MHC-I (BioLegend, catalog 116525) and MHC-I SIINFEKL (eBioscience, catalog 17-5743-82). The B16-OVA or ID8-OVA tumor cells were cocultured with T cells (B3Z or OT-I cells) for 24 hours. The LacZ activity and supernatant levels of IL-2 and IFN-γ were examined as previously described (40). For intracellular cytokine staining, GolgiStop reagent (1,000×; BD Biosciences) was added to the coculture system for 3 hours before staining. First, OT-I cells were stained with fluorescence-labeled antibodies against CD8 (BioLegend, catalog 100706) for 1 hour at 4°C. Next, the cells were fixed and permeabilized using an intracellular fixation and permeabilization buffer kit (eBioscience) and stained with anti–IFN-γ (eBioscience, catalog 17-7311-82) or anti-GZMB (eBioscience, catalog 12-8898-82). The stained cells were then analyzed using flow cytometry.

### OT-I T cell culture and cytotoxic T lymphocyte assay.

OT-I T cells were isolated from the spleen and lymph nodes of 8-week-old OT-I mice (purchased from The Jackson Laboratory) using MagniSort Mouse CD8 T-Cell Enrichment Kit (Thermo Fisher Scientific) according to the manufacturer’s guidance. OT-I T cells were maintained in complete RPMI 1640 (Gibco) supplemented with 2-mercaptoethanol (Gibco) or treated with SIINFEKL peptide (GenScript) for OT-I T cell activation. For cytotoxic T lymphocyte assay, the B16-OVA or ID8-OVA tumor cells were transfected with siNC or siRAD21 for 48 hours and then treated with or without recombinant IFN-β (R&D Systems) for another 24 hours. The pretreated cells were then cocultured with activated OT-I T cells at a ratio of 1:1 for 48 hours. Apoptotic cells were quantified using the Annexin V–FITC Apoptosis Detection Kit (Vazyme) according to the manufacturer’s protocol and analyzed with a BD LSRFortessa X-20 (BD Biosciences). The LDH release was determined using CytoTox96 Non-Radioactive Cytotoxicity Assay Kit (Promega) following the manufacturer’s instructions, and percentage cytotoxicity was calculated as per a previous study (59).

### In vivo studies.

Six- to eight-week-old female C57BL/6J and BALB/c nude mice were purchased from the Beijing Vital River Laboratory Animal Technology Company. All the mice were housed under specific pathogen–free conditions in the Laboratory Animal Center of Sun Yat-sen University. B16-OVA cells (5 × 10^5^) infected with scramble sgRNA or sgRAD21 were subcutaneously transplanted in the left flank of mice. Tumor volumes and body weights were monitored every 1–3 days after injection until tumor volume reached approximately 1,000–1,500 mm^3^. For the intraperitoneal model, ID8 cells (1 × 10^7^) were injected i.p. into C57BL/6 mice, and tumor progression was measured once a week using In Vivo Imaging System (Caliper Life Science). Tumor-bearing mice were treated by i.p. injection with control IgG or anti–PD-1 antibody (Bio X Cell, BE0273) at the indicated time points. These mice were sacrificed by CO_2_ inhalation, and their tumors were harvested for further analysis. For tumor-infiltrating CD8^+^ T cell analysis, commensurable mouse tumors were dissociated and filtered to generate single-cell suspensions; the cells were then stained with CD45 (BioLegend, catalog 103134), CD3 (BioLegend, catalog 100206), CD8 (BioLegend, catalog 100706), CD69 (BioLegend, catalog 104514), IFN-γ (eBioscience, catalog 17-7311-82), and GZMB (eBioscience, catalog 12-8898-82) and analyzed by flow cytometry.

### Data availability.

RNA-Seq and ChIP-Seq data were deposited in the NCBI’s Gene Expression Omnibus database (GEO GSE156845 and GSE193620).

### Statistics.

Data are presented as mean ± SD unless otherwise stated. Statistical significance of differences between 2 groups was evaluated by 2-tailed Student’s *t* test, while statistical significance of differences among multiple groups was analyzed by 1-way ANOVA or 2-way ANOVA using GraphPad Prism software. *P* values less than 0.05 were considered statistically significant.

### Study approval.

All animal studies were conducted in compliance with animal protocols approved by the Institutional Animal Care and Use Committee at Sun Yat-sen University Cancer Center. Archived patient samples were obtained from Sun Yat-sen University Cancer Center with approval from the medical ethics committee and signed patient informed consent.

## Author contributions

PD and JT conceived, designed, and supervised the study. PD performed the experiments. PD, PG, and ZZ analyzed and interpreted the data (e.g., statistical analysis, biostatistics, computational analysis). J Li, MZ, CZ, J Liu, YX, and QJ provided patient samples. Jieping Chen and WL provided pathology expertise. Shaoyan Liu and ZX performed the FISH experiments. Shini Liu, Jinghong Chen, and LL performed the ChIP experiments. ZW, XY, Shini Liu, ZY, YH, RX, JG, JH, JY, HL, Jianfeng Chen, and YW provided material and technical support and animal work. PD and JT wrote, reviewed, and revised the manuscript. XX provided key reagents and revised the manuscript. PZ, TK, QY, BTT, and JYC provided a kind suggestion for the manuscript writing. All the authors gave their consent to publish this study.

## Supplementary Material

Supplemental data

Supplemental table 1

Supplemental table 2

Supplemental tables 3-6

## Version 1 10/06/2022

In-Press Preview

## Version 2 11/15/2022

Electronic publication

## References

[1] Siegel RL (2021). Cancer statistics, 2021. CA Cancer J Clin.

[2] Wei SC (2018). Fundamental mechanisms of immune checkpoint blockade therapy. Cancer Discov.

[3] Halle S (2017). Mechanisms and dynamics of T cell-mediated cytotoxicity in vivo. Trends Immunol.

[4] Disis ML (2019). Efficacy and safety of avelumab for patients with recurrent or refractory ovarian cancer: phase 1b results from the JAVELIN solid tumor trial. JAMA Oncol.

[5] Bowtell DD (2015). Rethinking ovarian cancer II: reducing mortality from high-grade serous ovarian cancer. Nat Rev Cancer.

[6] Beroukhim R (2010). The landscape of somatic copy-number alteration across human cancers. Nature.

[7] Sarkar S (2017). PRKCI promotes immune suppression in ovarian cancer. Genes Dev.

[8] Kato S (2020). Gain-of-function genetic alterations of G9a drive oncogenesis. Cancer Discov.

[9] Griffin GK (2021). Epigenetic silencing by SETDB1 suppresses tumour intrinsic immunogenicity. Nature.

[10] Losada A (2014). Cohesin in cancer: chromosome segregation and beyond. Nat Rev Cancer.

[11] Waldman T (2020). Emerging themes in cohesin cancer biology. Nat Rev Cancer.

[12] Guo G (2013). Whole-genome and whole-exome sequencing of bladder cancer identifies frequent alterations in genes involved in sister chromatid cohesion and segregation. Nat Genet.

[13] Kon A (2013). Recurrent mutations in multiple components of the cohesin complex in myeloid neoplasms. Nat Genet.

[14] Solomon DA (2013). Frequent truncating mutations of STAG2 in bladder cancer. Nat Genet.

[15] Solomon DA (2011). Mutational inactivation of STAG2 causes aneuploidy in human cancer. Science.

[16] Nasmyth K, Haering CH (2009). Cohesin: its roles and mechanisms. Annu Rev Genet.

[17] Monahan K (2012). Role of CCCTC binding factor (CTCF) and cohesin in the generation of single-cell diversity of protocadherin-α gene expression. Proc Natl Acad Sci U S A.

[18] Guillou E (2010). Cohesin organizes chromatin loops at DNA replication factories. Genes Dev.

[19] Nitzsche A (2011). RAD21 cooperates with pluripotency transcription factors in the maintenance of embryonic stem cell identity. PLoS One.

[20] Galeev R (2016). Genome-wide RNAi screen identifies cohesin genes as modifiers of renewal and differentiation in human HSCs. Cell Rep.

[21] Fisher JB (2017). The cohesin subunit Rad21 is a negative regulator of hematopoietic self-renewal through epigenetic repression of Hoxa7 and Hoxa9. Leukemia.

[22] Yan J (2013). Transcription factor binding in human cells occurs in dense clusters formed around cohesin anchor sites. Cell.

[23] Xu H (2014). Cohesin Rad21 mediates loss of heterozygosity and is upregulated via Wnt promoting transcriptional dysregulation in gastrointestinal tumors. Cell Rep.

[24] Mazumdar C (2015). Leukemia-associated cohesin mutants dominantly enforce stem cell programs and impair human hematopoietic progenitor differentiation. Cell Stem Cell.

[25] Xu H (2011). Enhanced RAD21 cohesin expression confers poor prognosis and resistance to chemotherapy in high grade luminal, basal and HER2 breast cancers. Breast Cancer Res.

[26] Cancer Genome Atlas Research Network (2011). Integrated genomic analyses of ovarian carcinoma. Nature.

[27] Yamamoto Y (2016). In vitro and in vivo correlates of physiological and neoplastic human Fallopian tube stem cells. J Pathol.

[28] Karst AM (2014). Cyclin E1 deregulation occurs early in secretory cell transformation to promote formation of fallopian tube-derived high-grade serous ovarian cancers. Cancer Res.

[29] Bitler BG (2015). Synthetic lethality by targeting EZH2 methyltransferase activity in ARID1A-mutated cancers. Nat Med.

[30] Yang H (2004). Aurora-A kinase regulates telomerase activity through c-Myc in human ovarian and breast epithelial cells. Cancer Res.

[31] Győrffy B (2012). Implementing an online tool for genome-wide validation of survival-associated biomarkers in ovarian-cancer using microarray data from 1287 patients. Endocr Relat Cancer.

[32] Xia Y (2014). YAP promotes ovarian cancer cell tumorigenesis and is indicative of a poor prognosis for ovarian cancer patients. PLoS One.

[33] Tomikawa J (2020). Exploring trophoblast-specific Tead4 enhancers through chromatin conformation capture assays followed by functional screening. Nucleic Acids Res.

[34] Kim M (2015). Transcriptional co-repressor function of the hippo pathway transducers YAP and TAZ. Cell Rep.

[35] Bald T (2014). Immune cell-poor melanomas benefit from PD-1 blockade after targeted type I IFN activation. Cancer Discov.

[36] He Y (2021). FOXA1 overexpression suppresses interferon signaling and immune response in cancer. J Clin Invest.

[37] Li T (2020). TIMER2.0 for analysis of tumor-infiltrating immune cells. Nucleic Acids Res.

[38] Sistigu A (2014). Cancer cell-autonomous contribution of type I interferon signaling to the efficacy of chemotherapy. Nat Med.

[39] Rooney MS (2015). Molecular and genetic properties of tumors associated with local immune cytolytic activity. Cell.

[40] Wang Z (2019). cGAS/STING axis mediates a topoisomerase II inhibitor-induced tumor immunogenicity. J Clin Invest.

[41] Walton J (2016). CRISPR/Cas9-mediated Trp53 and Brca2 knockout to generate improved murine models of ovarian high-grade serous carcinoma. Cancer Res.

[42] Holmes D (2015). The problem with platinum. Nature.

[43] Patch AM (2015). Whole-genome characterization of chemoresistant ovarian cancer. Nature.

[44] Xu S (2016). miR-424(322) reverses chemoresistance via T-cell immune response activation by blocking the PD-L1 immune checkpoint. Nat Commun.

[45] Johnson R, Halder G (2014). The two faces of Hippo: targeting the Hippo pathway for regenerative medicine and cancer treatment. Nat Rev Drug Discov.

[46] Ni X (2018). YAP is essential for Treg-mediated suppression of antitumor immunity. Cancer Discov.

[47] Hornburg M (2021). Single-cell dissection of cellular components and interactions shaping the tumor immune phenotypes in ovarian cancer. Cancer Cell.

[48] Webb JR (2016). PD-L1 expression is associated with tumor-infiltrating T cells and favorable prognosis in high-grade serous ovarian cancer. Gynecol Oncol.

[49] Barkal AA (2019). CD24 signalling through macrophage Siglec-10 is a target for cancer immunotherapy. Nature.

[50] Ayers M (2017). IFN-γ-related mRNA profile predicts clinical response to PD-1 blockade. J Clin Invest.

[51] Subudhi SK (2020). Neoantigen responses, immune correlates, and favorable outcomes after ipilimumab treatment of patients with prostate cancer. Sci Transl Med.

[52] Borden EC (2019). Interferons α and β in cancer: therapeutic opportunities from new insights. Nat Rev Drug Discov.

[53] Ishizuka JJ (2019). Loss of ADAR1 in tumours overcomes resistance to immune checkpoint blockade. Nature.

[54] Davoli T (2017). Tumor aneuploidy correlates with markers of immune evasion and with reduced response to immunotherapy. Science.

[55] Segovia C (2019). Inhibition of a G9a/DNMT network triggers immune-mediated bladder cancer regression. Nat Med.

[56] Zhang Y (2021). MET amplification attenuates lung tumor response to immunotherapy by inhibiting STING. Cancer Discov.

[57] Oreskovic E (2022). Genetic analysis of cancer drivers reveals cohesin and CTCF as suppressors of PD-L1. Proc Natl Acad Sci U S A.

[58] Deb S (2014). RAD21 cohesin overexpression is a prognostic and predictive marker exacerbating poor prognosis in KRAS mutant colorectal carcinomas. Br J Cancer.

[59] Xu F (2021). Mevalonate blockade in cancer cells triggers CLEC9A^+^ dendritic cell-mediated antitumor immunity. Cancer Res.

[60] Camp RL (2004). X-tile: a new bio-informatics tool for biomarker assessment and outcome-based cut-point optimization. Clin Cancer Res.

[61] Yu Z (2021). Inhibition of the PLK1-coupled cell cycle machinery overcomes resistance to oxaliplatin in colorectal cancer. Adv Sci (Weinh).

[62] Liu S (2021). Targeting enhancer reprogramming to mitigate MEK inhibitor resistance in preclinical models of advanced ovarian cancer. J Clin Invest.

[63] Chen S (2018). fastp: an ultra-fast all-in-one FASTQ preprocessor. Bioinformatics.

[64] Dobin A (2013). STAR: ultrafast universal RNA-seq aligner. Bioinformatics.

[65] Li B, Dewey CN (2011). RSEM: accurate transcript quantification from RNA-Seq data with or without a reference genome. BMC Bioinformatics.

[66] Subramanian A (2005). Gene set enrichment analysis: a knowledge-based approach for interpreting genome-wide expression profiles. Proc Natl Acad Sci U S A.

[67] Langmead B, Salzberg SL (2012). Fast gapped-read alignment with Bowtie 2. Nat Methods.

[68] Feng J (2012). Identifying ChIP-seq enrichment using MACS. Nat Protoc.

[69] Ramirez F (2014). deepTools: a flexible platform for exploring deep-sequencing data. Nucleic Acids Res.

[70] Yu G (2015). ChIPseeker: an R/Bioconductor package for ChIP peak annotation, comparison and visualization. Bioinformatics.

[71] Thorvaldsdottir H (2013). Integrative Genomics Viewer (IGV): high-performance genomics data visualization and exploration. Brief Bioinform.

[72] Heinz S (2010). Simple combinations of lineage-determining transcription factors prime cis-regulatory elements required for macrophage and B cell identities. Mol Cell.

[73] Lee ST (2011). Context-specific regulation of NF-κB target gene expression by EZH2 in breast cancers. Mol Cell.

