# Supplementary material for: RAD21 amplification epigenetically suppresses interferon signaling to promote immune evasion in ovarian cancer
Source: J Clin Invest. 2022 Nov 15;132(22):e159628. doi: 10.1172/JCI159628 (PMC9663158; doi:10.1172/JCI159628)
Supplement: Supplemental data [file jci-132-159628-s185.pdf]

Supplemental Figure 1

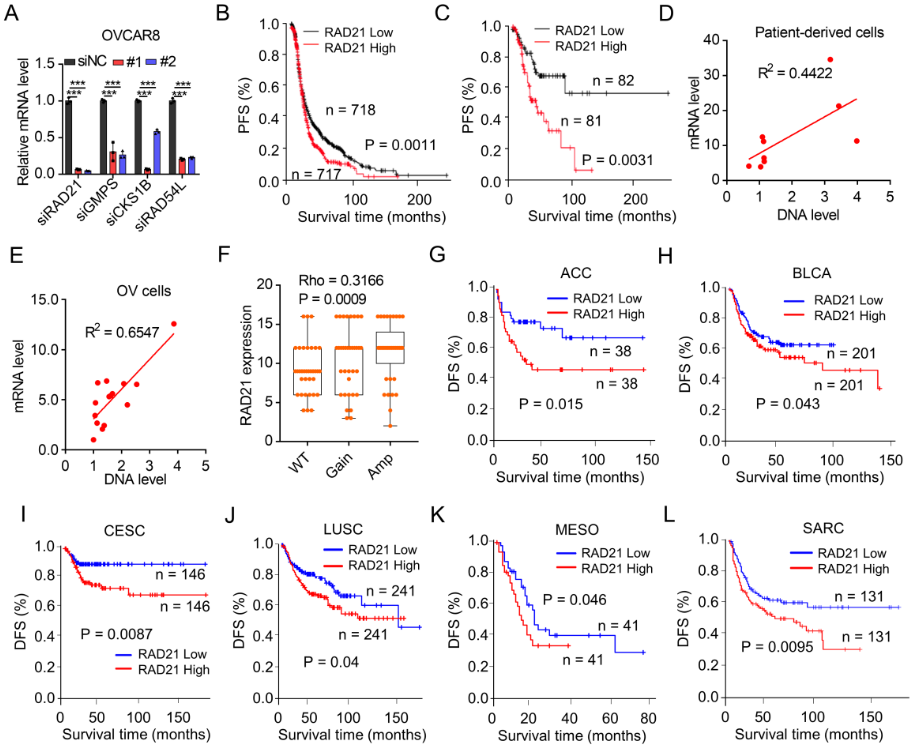

Figure S1. RAD21 is a critical CNA gene and amplified RAD21 correlates with poor prognosis in HGSOC. (A) qRT-PCR validation in OVCAR8 cells transfected with scramble siRNA or two individual siRNAs targeting *RAD21*, *GMPS*, *CKS1B* and *RAD54L*, respectively. Data are shown as mean ± SD (n = 3, one-way ANOVA). (B-C) Kaplan–Meier curves of the progression free survival rates in patients with OV grouped according to high and low expression of *RAD21*. (B) All OV patients and (C) Early-stage OV patients (Log-rank test). (D-E) The positive correlations between mRNA level (y-axis) and DNA level (x-axis) of *RAD21* in patient-derived cell lines (D) and commercial OV cell lines (E). DNA level were quantified with qRT-PCR using exon/intron primer. (F) Correlation analysis showing an increase in RAD21 mRNA level concordant with the gain of an additional DNA copy (Gain) and/or multiple copies (Amp) (SYSUCC-cohort, Spearman Rho = 0.3166; P = 0.0009). (G-L) Kaplan–Meier curves of Disease-Free Survival (DFS) in patients grouped according to high and low expression of RAD21. Data derived from the TCGA database. (ACC: Adrenocortical carcinoma, BLCA: Bladder Urothelial Carcinoma, CESC: Cervical squamous cell carcinoma and endocervical adenocarcinoma, LUSC: Lung Squamous Cell Carcinoma, MESO: Mesothelioma, SARC: Sarcoma) (Log-rank test).

Supplemental Figure 2

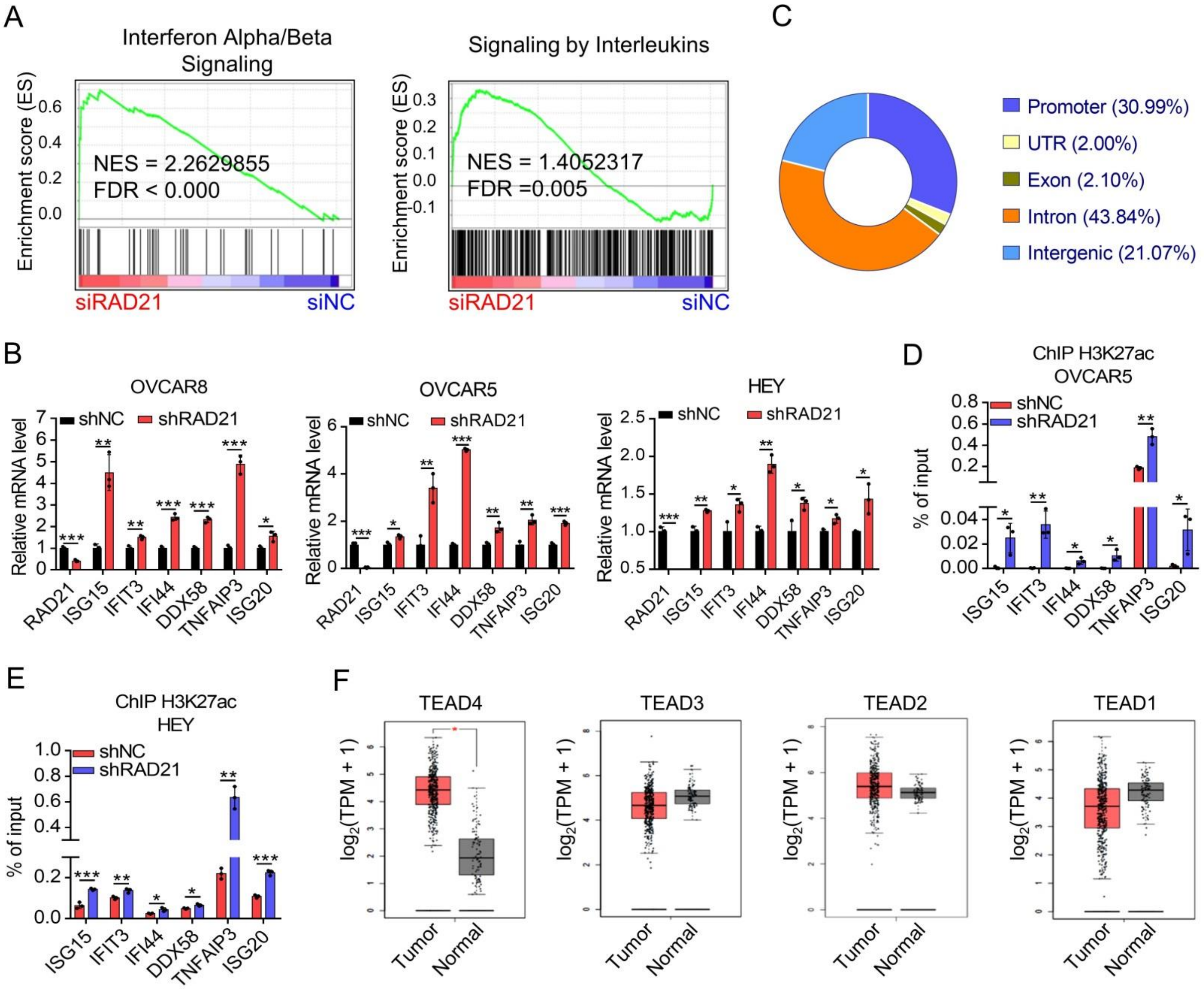

Figure S2. Genome-wide identification of transcriptional targets of RAD21 and its associated complexes

(A) GSEA analysis showing the Interferon Alpha/Beta Signaling and Signaling by Interleukins were enriched among the upregulated pathways. (B) qRT-PCR validation of representative ISGs in RAD21-KD and control OVCAR8, OVCAR5 and HEY cells mediated by shRNA. Data are shown as mean ± SD (n = 3, Two-tailed t-tests). (C) Genomic distribution of H3K27ac peaks in OVCAR8 cells. (D-E) ChIP-qPCR analysis of H3K27ac binding of representative ISGs in RAD21-depleted OVCAR5 (D) and HEY (E) cells. Data are shown as mean ± SD (n = 3, Two-tailed t-tests). (F) TEAD4, TEAD3, TEAD2, TEAD1 expression level in ovarian tumor and normal tissues as identified through the GEPIA website (Two-tailed t-tests , *, p < 0.01).

Supplemental Figure 3

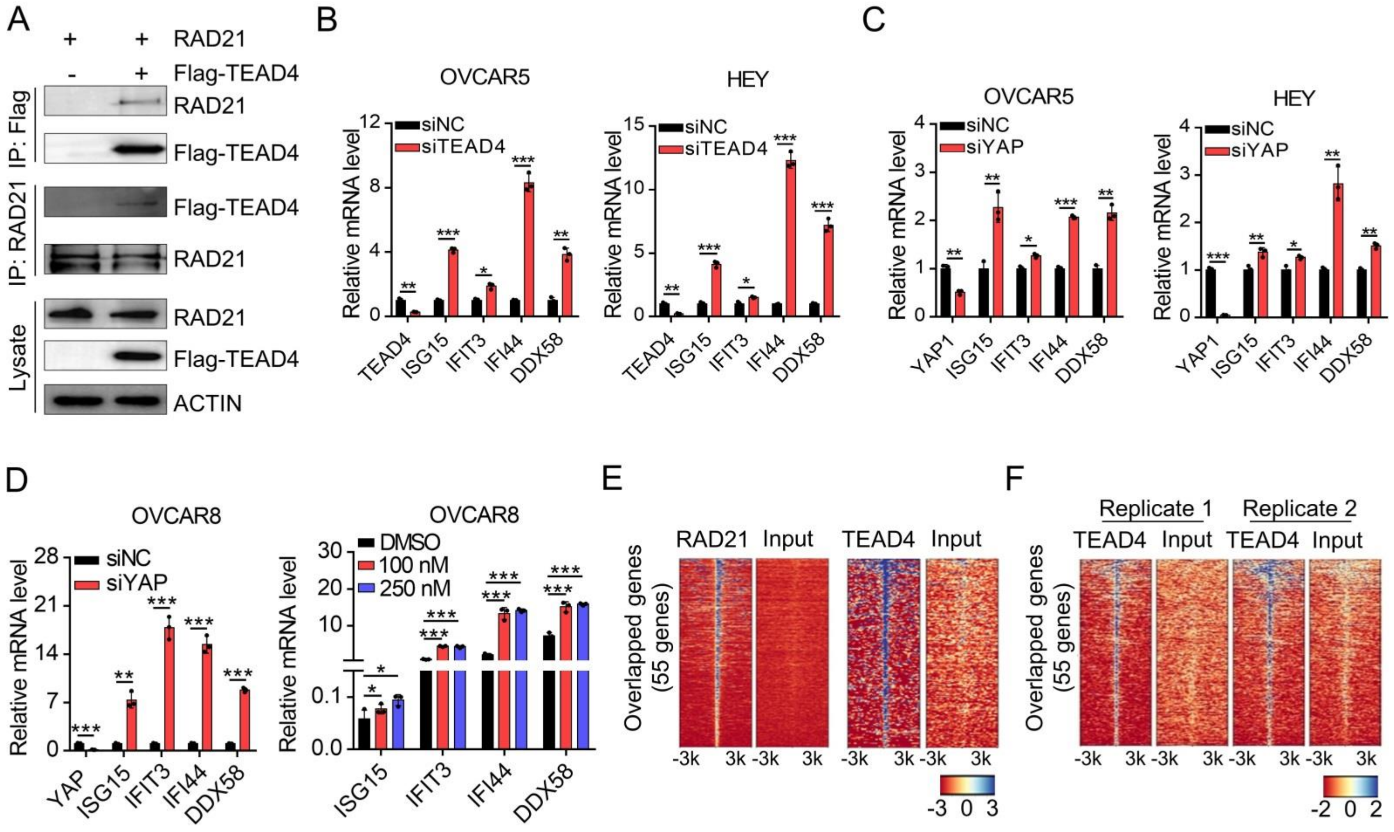

Figure S3. RAD21 directly interacts with YAP/TEAD4 transcriptional co-repressor complex to coordinately suppress ISGs

(A) Co-immunoprecipitation (Co-IP) shows the interaction of FLAG-tagged TEAD4 and RAD21 in 293T cells. (B-C) qRT-PCR validation of representative ISGs in *TEAD4*-KD (B) or *YAP*-KD (C) and control OVCAR5 and HEY cells. Data are shown as mean ± SD (n = 3, Two-tailed t-tests). (D) qRT-PCR validation of representative ISGs in OVCAR8 cells treated with vehicle and siYAP (left) or verteporfin (right) (100 nM and 250 nM). Data are shown as mean ± SD (n = 3, Two-tailed t-tests (left) and one-way ANOVA (right)). (E) Heatmap showing the binding patterns for TEAD4 at accessible regions of genes described in Figure 3G. (F) Heatmap showing the binding patterns for TEAD4 ChIP-seq peaks (obtained from ENCODE, GSM1010845, duplicates) at accessible regions of genes described in Figure 3G. *, $P < 0.05$; **, $P < 0.01$; ***, $P < 0.001$; n.s, no significance.

Supplemental Figure 4

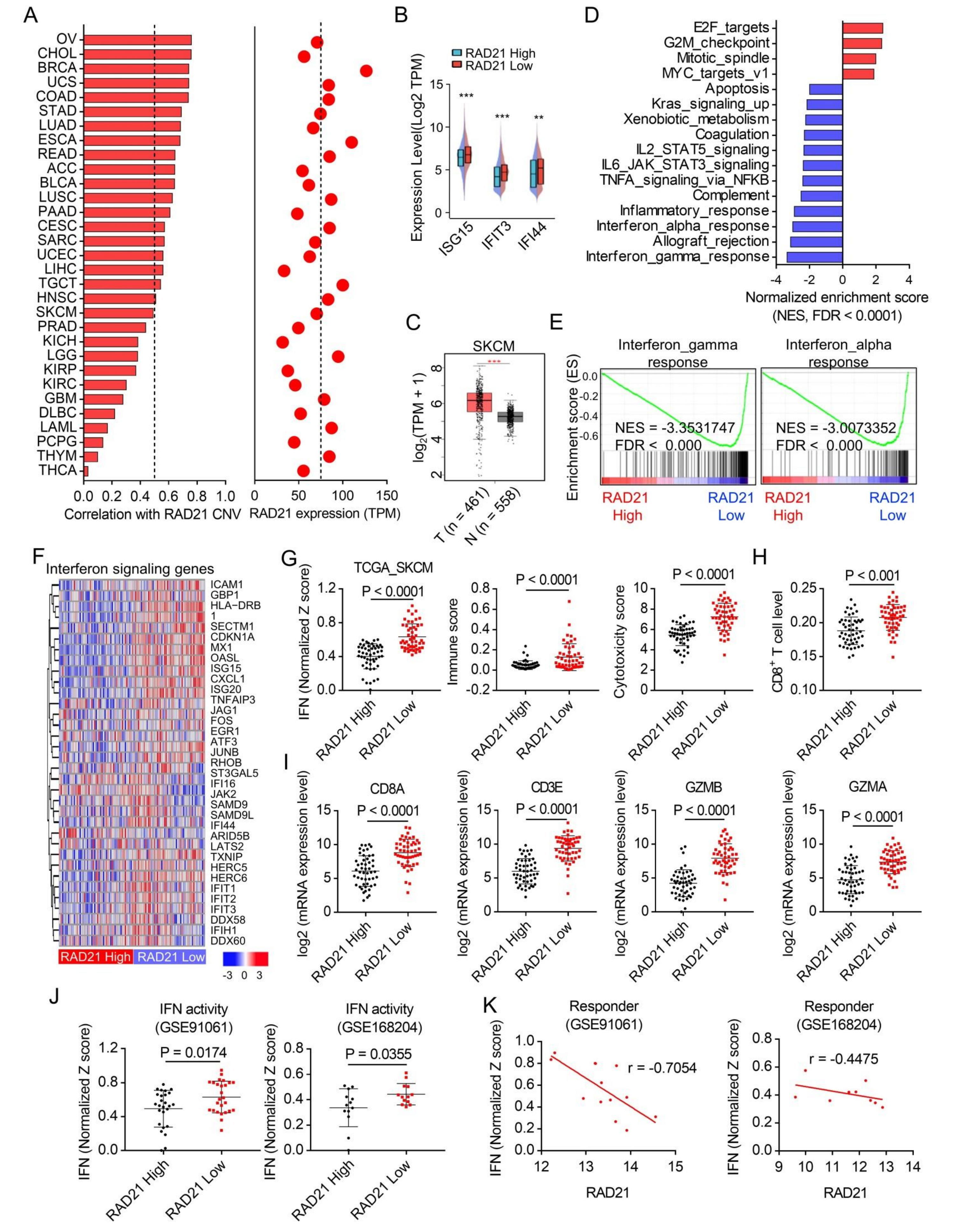

Figure S4. RAD21 inversely correlates with IFN signalling activity in SKCM

(A) Spearman's correlation between RAD21 copy number and mRNA (left), and cases with RAD21 high- or low-level expression (right) in TCGA database. Dotted lines indicate mean values. (B) mRNA levels of *ISG15*, *IFIT3* and *IFI44* in a diverse panel of 1,377 cancer cell lines from the CCLE (Two-tailed t-tests). Tumor cell lines were grouped based on RAD21 expression level. (C) The comparative expression level (TPM) of RAD21 in 461 skin cutaneous melanoma tissues and 558 normal tissues based on TCGA data using the GEPIA website. (***. $p<0.001$). (D) Normalized enrichment scores correlated with RAD21 expression using Hallmark gene sets in TCGA-SKCM database. High vs. low RAD21 expression (top vs. bottom 10%) (n=50/group, FDR, false-discovery rate. (E-F) GSEA analysis (E) and heatmap (F) showing the inverse correlation between RAD21 expression and the interferon signalling pathways and genes in TCGA-SKCM database. (G-I) Comparative analysis showing the association of high expression of RAD21 with low expression of IFN activity, immune score, cytotoxicity score (G), infiltration levels of $CD8^{+}$ (H) and T cell marker genes CD8A, CD3E, GZMA and GZMB (I) in patients with SKCM from TCGA database (Two-tailed t-tests). (J-K) Negative correlation between RAD21 level and IFN activity in all patients (J) and in responders (K) from GSE91061 and GSE168204 datasets (Pearson's correlation coefficient).

Supplemental Figure 5

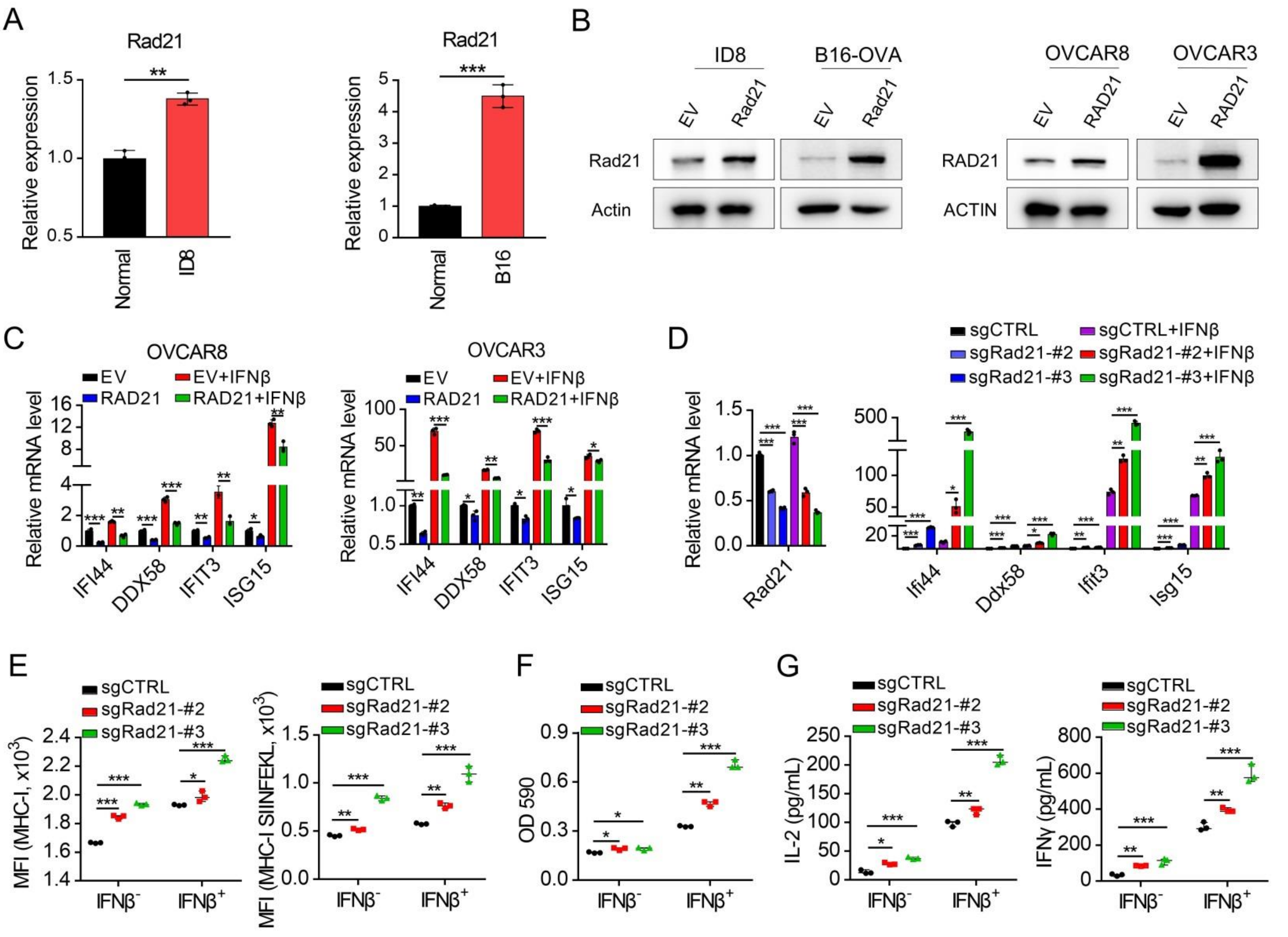

Figure S5. RAD21 ablation induces T cell activation *in vitro*.

(A) Relative mRNA level of Rad21 in mouse ovarian cancer cell line ID8 and mouse skin cutaneous melanoma cell line B16. (Mouse skin and ovary tissues were used as normal control individually, bars represent mean ± SD, n = 3). (B) Immunoblot analysis of indicated proteins in OVCAR8, OVCAR3, B16-OVA and ID8 cells with stable expression of empty vector (EV) or RAD21. (C) Relative mRNA level of representative ISGs (IFI44, DDX58, IFIT3 and ISG15) in RAD21-overexpressed and control OVCAR8 and OVCAR3 cells in the presence or absence of IFN-β treatment. (D) qRT-PCR validation of representative ISGs *Ifi44*, *Ddx58*, *Ifit3* and *Isg15* in *Rad21*-KO and control B16-OVA mediated by sgRNAs in the presence or absence of IFN-β treatment. Data are shown as mean ± SD (n = 3, one-way ANOVA). (E) Expression levels of MHC-I and MHC-I–SIINFEKL on *Rad21*-KO and control B16-OVA cells treated with vehicle or IFN-β. (MFI, mean fluorescence intensity, data are shown as mean ± SD, n = 3, one-way ANOVA). (F-G) *Rad21*-KO and control B16-OVA cells were treated with vehicle or IFN-β and then cocultured with B3Z cells or OT-I cells, after which B3Z activation was determined by LacZ activity (F) and OT-I activation was determined by secretion of IL-2 and IFN-γ (G). Data are shown as mean ± SD (n = 3 , one-way ANOVA).

Supplemental Figure 6

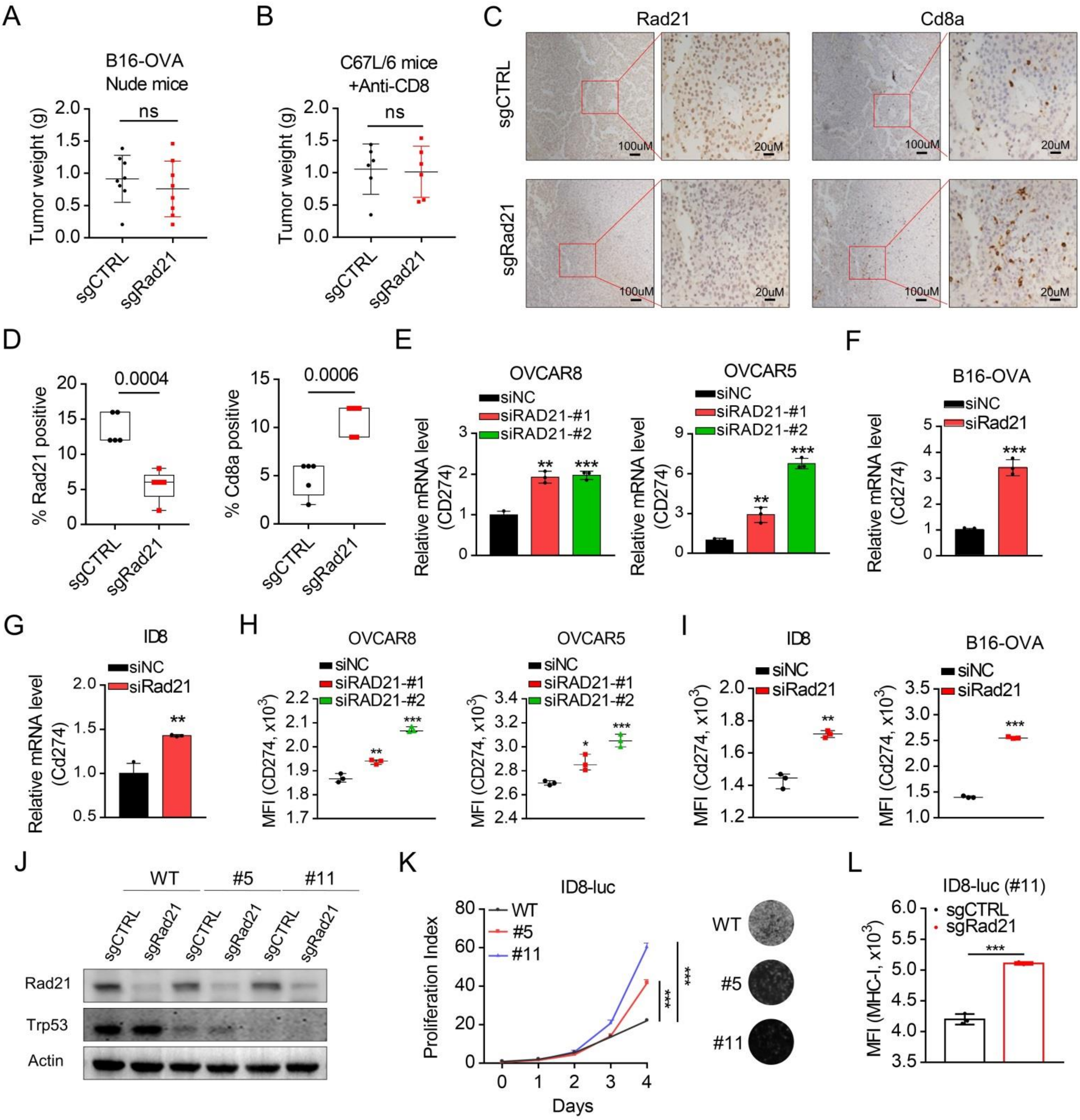

Figure S6. RAD21 suppresses antitumor immunity in vivo

(A-B) Tumor weight in nude mice and C57BL/6 mice described in Figure 6B-6C. Data are shown as mean ± SD (Nude mice, n = 8; C57BL/6 mice, n = 6, Two-tailed t-tests). (C-D) Representative IHC staining (C) and quantification showing the expression of Rad21 and Cd8a (D) in tumors described in Figure 6D-6E (Two-tailed t-tests). (E-I) qRT-PCR (E-G) and FACS analysis (H-I) of the expression level of PD-L1 (CD274) in OVCAR8, OVCAR5, ID8 and B16-OVA cells transfected with scramble siRNA or siRNAs targeting RAD21. (MFI, mean fluorescence intensity). (J) Immunoblot analysis of showing knockout efficacy of Trp53 or Rad21 mediated by sgRNAs in polyclonal ID8 cells with stable lentiviral sgCTRL and sgTrp53 (#5/#11). (K) Growth curve and colony formation are shown in polyclonal ID8 cells with Trp53 knock out. (Two-way ANOVA). (L) Expression level of MHC-I in Rad21-knockout and control $ID8^{Trp53-/-}$ cells. (MFI, Mean Fluorescence Intensity, Two-tailed t-tests).
