# Supplementary material for: RAD21 amplification epigenetically suppresses interferon signaling to promote immune evasion in ovarian cancer
Source: J Clin Invest. 2022 Nov 15;132(22):e159628. doi: 10.1172/JCI159628 (PMC9663158; doi:10.1172/JCI159628)
Supplement: Supplemental tables 3-6 [file jci-132-159628-s188.pdf]

Supplemental Tables

Supplemental Table 1: Copy number alterations form TCGA 572 samples.

Supplemental Table 2: RAD21 direct targets.

Supplemental Tables 3-6. Patient characteristics and sequences of siRNA, qRT-PCR and ChIP-qPCR primers.

Supplemental Table 1 and Table 2 are available as excel files.

Supplemental Table 3. Patient characteristics.

**Characteristic**	**All cases**
**Number of patients**	107
**Age at the time of surgery, years**	52.99± 9.43
**FIGO staging, n (%)**	
I	13 (12.1%)
II	22 (20.6%)
III	58 (54.2%)
IV	14(13.1%)
**Adjuvant chemotherapy, n (%)**	107 (100%)
**Chemotherapy regimens, n (%)**	
TP (Paclitaxel + Carboplatin)	106 (99.0%)

DP (Docetaxel + Carboplatin)	1 (1.0%)
**Median follow-up time, months**	49.65

Supplemental Table 4. The sequences of siRNAs, shRNA, sgRNAs and primers for genes cloning.

**Identifier**	**Sequences (5' - 3')**
siNC	UUCUCCGAACGUGUCACGUTT
si*RAD21*-#1 (1)	CTCCAAATATCTGTCAGCTAA
si*RAD21*-#2 (2)	ATCGATGAGCCCATTATTGAA
si*GMPS*-#1	GCATTTGCTATAAAGGAACAA
si*GMPS*-#2	GGGAAAGUCAUAGACCGAATT
si*CKS1B*-#1	GGAGGAAGCAUCUGAGUUUTT
si*CKS1B*-#2	GGAGUUUGAGUAUCGACAUTT
si*RAD54L*-#1	CTTTGTAATCATCCAGCTCTA
si*RAD54L*-#2	CCAGAGTGCAAGCCAGAAATT
si*YAP* (3)	GACAUCUUCUGGUCAGAGAdTdT
si*TEAD4* (4)	GGAACAAACUGUGCCUGAATT
sh*RAD21*-#1	CTCCAAATATCTGTCAGCTAA
sh*RAD21*-inducible	CCAGATAGCATCAGAAATCAT
sg*Rad21*-#2(mouse)	GCAGTGGCTGATCAAAATCG
sg*Rad21*-#3(mouse)	GTAACCGATGACATCTCGCTG

si*Rad21* (mouse) (5)	GCGGUAUAUUAGAUGACAATT
RAD21-F	ATGTTCTACGCACATTTTGTTC
RAD21-R	TTATATAATATGGAACCTTG
TEAD4-F	TTGGAGGGCACGGCCGGCA
TEAD4-R	TCATTCTTTCACCAGCCTG
Rad21-F	ATGTTCTACGCACATTTTGT
Rad21-R	TCAGATAATATGGAACCGTG

Supplemental Table 5. Quantitative Real-Time PCR (qRT-PCR) Primers.

Genes	Forward (5' - 3')	Reverse (5' - 3')
18S	GTAACCCGTTGAACCCCATT	CCATCCAATCGGTAGTAGCG
RAD54L	AGGCAGGTCCTGTGATGATGA	TCAAAGGTTTCCGAAAAGGAGA
CKS1B	CGGACAAATACGACGACGAGG	CTCTGCTGAACGCCAAGATTCC
GMPS	CAAGGTTGTGGCACGTTCTG	GCACGGTGAAGGTTCCACT
RAD21	AAGCCAAATACCTTCTTGCAGAC	CTGCTTCCCGATTTTCCTCAG
YAP	TAGCCCTGCGTAGCCAGTTA	TCATGCTTAGTCCACTGTCTGT
TEAD4	GGACACTACTCTTACCGCATCC	TCAAAGACATAGGCAATGCACA
ISG15	GCGCAGATCACCCAGAAGAT	TCCTCACCAGGATGCTCAGA
TNFAIP3	TCCTCAGGCTTTGTATTTGAGC	TGTGTATCGGTGCATGGTTTTA
ISG20	CTCGTTGCAGCCTCGTGAA	CGGGTTCTGTAATCGGTGATCTC
IFIT3	AAAAGCCCAACAACCCAGAAT	CGTATTGGTTATCAGGACTCAGC
IFI44	ATGGCAGTGACAACTCGTTTG	TCCTGGTAACTCTCTTCTGCATA

DDX58	CTGGACCCTACCTACATCCTG	GGCATCCAAAAAGCCACGG
g-*RAD21*	GCCTGTGATGCCTGTAAG	CCACTTGCTACTGACTGTATG
g-*GAPDH*	CCGGGTTCATAACTGTCTGCTTC	TGGTTCACACCCATGACGAACA
Rad21	GCCCATGTATTTGAGTGCAA	ACTCCCAGGAGAAGGTGTCC
Isg15	GGTGTCCGTGACTAACTCCAT	TGGAAAGGGTAAGACCGTCCT
Ifi44	AACTGACTGCTCGCAATAATGT	GTAACACAGCAATGCCTCTTGT
Ifit3	CCTACATAAAGCACCTAGATGGC	ATGTGATAGTAGATCCAGGCGT
Ddx58	AAGAGCCAGAGTGTCAGAATCT	AGCTCCAGTTGGTAATTTCTTGG
Actin	AGAGGGAAATCGTGCGTGAC	CAATAGTGATGACCTGGCCGT

Supplemental Table 6. ChIP-qPCR Primers.

Genes	Forward (5' - 3')	Reverse (5' - 3')
ISG15	AGTCCATGTGATGACAGCTTTA	CAGAGTGAGCGGAAGAAACA
IFIT3	GGTGTACACACAGGCTTCTT	GCCAAGCCTTCCTCTCATT
TNFAIP3	CAACCCGCATACAACTGAAAC	GGGTGTGATCTCTCTTGGC
IFI44	CCCTCTGACTTCAATTCCTTGAT	GACAGCATGCCAACTTCTCT
ISG20	CCCTGACTTCACTTGATAACAAAC	CAGCGGTGACTCATCTCTAAC
DDX58	GTGCCAGGGTAAGTCTATGTTTA	ATCTACTGGAGCTCTCCTACTG

REFERENCES

1. Mahmood SF, Gruel N, Chapeaublanc E, Lescure A, Jones T, Reyal F, et al. A siRNA screen identifies RAD21, EIF3H, CHRAC1 and TANC2 as driver genes within the 8q23, 8q24.3 and 17q23 amplicons in breast cancer with effects on cell growth, survival and transformation. *Carcinogenesis.* 2014;35(3):670-82.
2. Kong X, Ball AR, Jr., Pham HX, Zeng W, Chen HY, Schmiesing JA, et al. Distinct functions of human cohesin-SA1 and cohesin-SA2 in double-strand break repair. *Molecular and cellular biology.* 2014;34(4):685-98.
3. Xia Y, Chang T, Wang Y, Liu Y, Li W, Li M, et al. YAP promotes ovarian cancer cell tumorigenesis and is indicative of a poor prognosis for ovarian cancer patients. *PLoS One.* 2014;9(3):e91770.
4. Jiao S, Li C, Hao Q, Miao H, Zhang L, Li L, et al. VGLL4 targets a TCF4-TEAD4 complex to coregulate Wnt and Hippo signalling in colorectal cancer. *Nat Commun.* 2017;8:14058.
5. Mullenders J, Aranda-Orgilles B, Lhoumaud P, Keller M, Pae J, Wang K, et al. Cohesin loss alters adult hematopoietic stem cell homeostasis, leading to myeloproliferative neoplasms. *J Exp Med.* 2015;212(11):1833-50.
